# Mendelian randomization analysis of maternal coffee consumption during pregnancy on offspring neurodevelopmental difficulties in the Norwegian Mother, Father and Child Cohort Study (MoBa)

**DOI:** 10.1017/S0033291724002216

**Published:** 2024-09

**Authors:** Shannon D'Urso, Robyn E Wootton, Helga Ask, Caroline Brito Nunes, Ole A Andreassen, Liang-Dar Hwang, Gunn-Helen Moen, David M Evans, Alexandra Havdahl

**Affiliations:** 1 Institute for Molecular Bioscience, The University of Queensland, Brisbane, Queensland, Australia; 2 MRC (Medical Research Council) Integrative Epidemiology Unit, University of Bristol, Bristol, UK; 3 School of Psychological Science, University of Bristol, Bristol, UK; 4 Nic Waals Institute, Lovisenberg Diaconal Hospital, Oslo, Norway; 5 PsychGen Center for Genetic Epidemiology and Mental Health, Norwegian Institute of Public Health, Oslo, Norway; 6 PROMENTA Research Center, Department of Psychology, University of Oslo, Oslo, Norway; 7 NORMENT Centre, Division of Mental Health and Addiction, Institute of Clinical Medicine, University of Oslo, Oslo University Hospital, Oslo, Norway; 8 Institute of Clinical Medicine, Faculty of Medicine, University of Oslo, Oslo, Norway; 9 K. G. Jebsen Center for Genetic Epidemiology, Department of Public Health and Nursing, NTNU, Norwegian University of Science and Technology, Trondheim, Norway; 10 Frazer Institute, The University of Queensland, Woolloongabba, Queensland, Australia; 11 The University of Queensland, Brisbane, Queensland, Australia

**Keywords:** ADHD, autism, caffeine, coffee, genetic epidemiology, Mendelian randomization, neurodevelopment

## Abstract

**Background:**

Previous observational epidemiological studies have suggested that coffee consumption during pregnancy may affect fetal neurodevelopment. However, results are inconsistent and may represent correlational rather than causal relationships. The present study investigated whether maternal coffee consumption was observationally associated and causally related to offspring childhood neurodevelopmental difficulties (NDs) in the Norwegian Mother, Father and Child Cohort Study.

**Methods:**

The observational relationships between maternal/paternal coffee consumption (before and during pregnancy) and offspring NDs were assessed using linear regression analyses (*N* = 58694 mother-child duos; *N* = 22 576 father-child duos). To investigate potential causal relationships, individual-level (*N* = 46 245 mother-child duos) and two-sample Mendelian randomization (MR) analyses were conducted using genetic variants previously associated with coffee consumption as instrumental variables.

**Results:**

We observed positive associations between maternal coffee consumption and offspring difficulties with social-communication/behavioral flexibility, and inattention/hyperactive-impulsive behavior (multiple testing corrected *p* < 0.005). Paternal coffee consumption (negative control) was not observationally associated with the outcomes. After adjusting for potential confounders (smoking, alcohol, education and income), the maternal associations attenuated to the null. MR analyses suggested that increased maternal coffee consumption was causally associated with social-communication difficulties (individual-level: beta = 0.128, se = 0.043, *p* = 0.003; two-sample: beta = 0.348, se = 0.141, *p* = 0.010). However, individual-level MR analyses that modelled potential pleiotropic pathways found the effect diminished (beta = 0.088, se = 0.049, *p* = 0.071). Individual-level MR analyses yielded similar estimates (heterogeneity *p* = 0.619) for the causal effect of coffee consumption on social communication difficulties in maternal coffee consumers (beta = 0.153, se = 0.071, *p* = 0.032) and non-consumers (beta = 0.107, se = 0.134, *p* = 0.424).

**Conclusions:**

Together, our results provide little evidence for a causal effect of maternal coffee consumption on offspring NDs.

## Introduction

Coffee is consumed widely within most societies around the world (International Coffee Council, 2012). Scandinavian countries rank highly in terms of caffeine intake, with an average daily intake of >400 mg of caffeine (equivalent to approximately four cups of coffee) (Fredholm, Bättig, Holmén, Nehlig, & Zvartau, 1999). During pregnancy, coffee consumption remains widespread (Fredholm et al., 1999; Lukic, Barnung, Skeie, Olsen, & Braaten, 2020). However, a vast array of physiological changes during gestation result in a significant reduction in maternal caffeine metabolism (Aldridge, Bailey, & Neims, 1981; Horning, Butler, Nowlin, & Hill, 1975; Soyka, 1981). Caffeine and its primary metabolites (paraxanthine, theophylline, and theobromine) are known to readily cross the placenta (Fernandes et al., 1998; Goldstein & Warren, 1962), and are not easily cleared due to underdevelopment of fetal caffeine metabolizing enzymes (Aldridge et al., 1981; Grosso & Bracken, 2005). Consequently, it has been proposed that accumulated caffeine metabolites may exert detrimental effects on the developing fetal brain (Brent, Christian, & Diener, 2011; Gressens, Mesples, Sahir, Marret, & Sola, 2001; Qian, Chen, Ward, Duan, & Zhang, 2020; Ross, Graham, Money, & Stanwood, 2014; Temple et al., 2017).

Several observational epidemiological studies have investigated the relationship between maternal coffee/caffeine consumption during pregnancy and offspring neurodevelopment; however, they have yielded inconsistent results (James, 2021). Many have found that increased maternal caffeine consumption is associated with offspring neurodevelopmental difficulties (NDs) (Hvolgaard Mikkelsen, Obel, Olsen, Niclasen, & Bech, 2017; Nishihara et al., 2022; Patti et al., 2021; Zhang, Manza, & Volkow, 2022), and one study reported that the effects differed according to caffeine source (i.e. an effect for soft drink but not tea or coffee) (Bekkhus, Skjøthaug, Nordhagen, & Borge, 2010). However, a number of other observational studies have reported no meaningful relationship between maternal coffee consumption during pregnancy and offspring NDs (Berglundh et al., 2021; Klebanoff & Keim, 2015; Linnet et al., 2009; Loomans et al., 2012).

Observational epidemiological studies of maternal coffee/caffeine consumption during pregnancy and offspring neurodevelopment are unlikely to sufficiently control for environmental and genetic confounders. Coffee and caffeine consumption are strongly positively associated with potential confounders such as age, smoking status, alcohol consumption, and negatively with maternal educational attainment (Brice & Smith, 2002; Hewlett & Smith, 2006; Papadopoulou et al., 2018; Torvik et al., 2020; Treur et al., 2016). Even when these confounders are accounted for, reporting biases and measurement error can render attempts at adjustment insufficient. For example, it has been proposed that while coffee consumption may be accurately reported, stigmatized behaviors such as smoking and drinking alcohol, especially during pregnancy, may be underreported and therefore adjustment may only partially remove their confounding effect (Cornelis & Munafo, 2018). Additionally, genetic confounding may also be an issue for observational studies– where for example, maternal coffee consumption, smoking, and/or alcohol consumption could be linked to maternal neurodevelopmental or psychiatric traits (e.g. ADHD, impulsivity, or anxiety), whose genetic predisposition can then be transmitted to offspring and influence their neurodevelopment (Havdahl et al., 2022; Hvolgaard Mikkelsen et al., 2017).

The Mendelian randomization (MR) method is robust to some of the issues faced by traditional observational epidemiological approaches (Davey Smith & Ebrahim, 2003). Following the identification of several genetic variants associated with coffee consumption (Cornelis et al., 2015) and caffeine metabolites (Cornelis et al., 2016) in genome-wide association studies (GWAS), MR has been used to investigate the causal effect of coffee/caffeine exposure, outside of and during pregnancy, on both own and offspring outcomes (Brito Nunes et al., 2022; Cornelis & Munafo, 2018). This includes the potential causal relationship between maternal caffeine consumption and offspring NDs (Haan et al., 2022; Schellhas et al., 2021). Schellhas and colleagues used maternal polygenic scores (PGS) to assess the causal relationship between maternal caffeine consumption (and smoking) on offspring neuropsychiatric outcomes (autism diagnosis, behavioral problems, and emotional problems across childhood and adolescence) in ~ 7000 mother-child duos in the Avon Longitudinal Study of Parents and Children (ALSPAC) (Schellhas et al., 2021). They found little evidence of an intrauterine (or offspring mediated) causal effect of maternal caffeine consumption on offspring outcomes. In addition, Haan and colleagues analyzed both ALSPAC (N ~ 8196 mothers) and the Norwegian Mother, Father and Child Cohort Study (early MoBa genetic data release; N ~ 14 584 mothers) and found no strong evidence for a causal effect of maternal smoking, alcohol or caffeine consumption (proxied by maternal PGS) and childhood ADHD symptoms at age 7–8 years (Haan et al., 2022).

In this study, we leveraged the world's largest cohort of genotyped parent-offspring trios, the Norwegian Mother, Father and Child Cohort Study (MoBa) (Magnus et al., 2016), to investigate the relationship between maternal coffee consumption and a broad range of offspring childhood NDs (46 245 mother-child duos with genetic and phenotypic data available). Firstly, we assessed the observational relationship between maternal coffee consumption (at three time points; before pregnancy, at week 15 and week 22) and offspring NDs, and implemented a paternal-negative control analysis to evaluate the likelihood of postnatal confounding effects (i.e. paternal coffee consumption should not be independently related to offspring neurodevelopment through prenatal pathways). Next, we used a range of MR approaches to investigate whether the observational relationships were likely to be causal. We leveraged the parent-child relationships within MoBa to control for offspring and paternal mediated pleiotropy (i.e. genetic confounding and postnatal mechanisms respectively). We assessed other potential sources of pleiotropy (smoking, alcohol consumption and education) using a series of MR sensitivity analyses. Finally, we conducted a gene by environment (GxE) MR analysis (Chen, Davey Smith, Harbord, & Lewis, 2008; Davey Smith, 2010), where we do not expect to see an effect of maternal genotype on offspring outcomes in mothers who reported no coffee consumption, if there is a true causal effect.

## Methods

### Cohort description

MoBa is a population-based pregnancy cohort study conducted by the Norwegian Institute of Public Health (Magnus et al., 2016). Participants were recruited from all over Norway from 1999–2008. All pregnant women in Norway during this period were eligible for participation in MoBa, resulting in a total of 277 702 invitations sent (Magnus et al., 2016, 2006). The women consented to participation in 41% of the pregnancies. Blood samples were obtained from both parents during pregnancy and from mothers and children (umbilical cord) at birth (Paltiel et al., 2014). The cohort includes approximately 114 500 children, 95 200 mothers and 75 200 fathers. The current study is based on version 12 of the quality-assured data files released for research in January 2019. MoBa has been linked to the Medical Birth Registry of Norway (MBRN), a national health registry containing information about all births in Norway. The establishment of MoBa and initial data collection was based on a license from the Norwegian Data Protection Agency and approval from The Regional Committees for Medical and Health Research Ethics. The MoBa cohort is currently regulated by the Norwegian Health Registry Act. The current study was approved by The Regional Committees for Medical and Health Research Ethics (2016/1702). This project used MoBa genetic data that was cleaned and imputed as per the MoBaPsychGen pipeline (*N* = 207 569 individuals passed quality control) (Corfield et al., 2022).

### Phenotype derivation

MoBa mothers completed ratings of offspring NDs at age 6, 18 and 36 months, 5 and 8 years. The *phenotools* R package (Hannigan et al., 2023) (v 0.2.2) was used to extract scale scores from ND measures across the MoBa questionnaires, covering a range of domains: language, motor, social communication, behavioral flexibility, attention and hyperactivity. See online Supplementary Materials 1 for a description of the 20 offspring ND measures that were extracted. Mean imputation was used to impute an individual's missing phenotype, when the number of non-missing items for that individual was greater than or equal to 50%. Items were reverse-coded where necessary so that high scores reflected greater difficulties. NDs were rank-based inverse normal transformed.

Three maternal coffee consumption variables were derived from self-reported dietary intake variables in MoBa Questionnaire 1 (administered at gestational week 15; reports of coffee consumption before pregnancy and at week 15) and Questionnaire 2 (week 22). For each timepoint, maternal coffee consumption (cups/day) was calculated from reported intake of filtered, instant and boiled coffees as well as lattes and espressos. Paternal coffee consumption (cups/day) was also derived from self-reports in the Father's Questionnaire (week 15). Maternal and paternal caffeine intake (mg/day) were also calculated using an approach previously described in a MoBa study (Papadopoulou et al., 2018). In short, multiple caffeine intake variables (mg/day; before pregnancy, week 15 and week 22) were derived by aggregating caffeine from all sources available in the questionnaires (several types of coffee, black and herbal tea, soft drink, and energy drinks) (Papadopoulou et al., 2018). Individuals who had consumed greater than 3.5L of coffee per day were excluded from all analyses (Papadopoulou et al., 2018).

Maternal smoking (cigarettes/week) was available at three timepoints (the last 3 months before pregnancy, at week 15 and week 30). Binary smoking (yes/no) variables were also derived for each time point. Maternal alcohol frequency (weekly) was assessed at five timepoints by the question ‘How often did you consume alcohol’ (i.e. times per week; last 3 months before pregnancy; week 0–12 (referred to as week 12), week 15; week 13–24 (referred to as week 24); and week 25 + (referred to as week 30)). Binary alcohol consumption (yes/no) variables were derived for each time point. Socioeconomic variables were extracted from Questionnaire 1. The highest level of education completed by both mothers and fathers was transformed into years of education according to the International Standard Classification of Education (ST1). Maternal and paternal gross yearly incomes in Norwegian Kroner (NOK) were also obtained, and each income bracket was converted to its respective midpoint (ST1). We note that all analyses involving income also included birth year, to account for some of the differing effects of income over time.

Offspring were excluded from analyses if they had congenital birth defects, chromosomal abnormalities, were a part of a multiple birth, as registered in MBRN, or identified as twins found in the KING analysis (Corfield et al., 2022; Manichaikul et al., 2010). Individuals who had withdrawn from MoBa at the time of analysis commencement (December 2022) were excluded.

### Multiple testing correction threshold

Since the neurodevelopmental trait measures are correlated, we used a principal component analysis (PCA) to estimate the effective number of statistical tests being performed and employed this number in statistical correction for multiple testing. The PCA was performed across 15 719 offspring with complete ND phenotypes available to determine the number of principal components (PCs) that explained >80% of the variance shared between the 20 neurodevelopmental outcomes, consistent with previous research (Havdahl et al., 2022). Ten PCs accounted for 80% of the covariance between traits, suggesting an approximate multiple-testing corrected *p*-value threshold of *p* < 0.005 for statistical significance in analyses involving all 20 ND outcomes (i.e. the traditional epidemiological analyses, individual-level MR and two-sample MR analyses). The sensitivity analyses, which were conducted across only one ND, used a less stringent *p*-value threshold of *p* < 0.05. Analyses were performed in R using the prcomp function.

### Traditional epidemiological analyses

Linear regression analyses were performed to investigate the relationship between the exposure, maternal coffee consumption at three time points (before pregnancy, week 15, week 22), and offspring NDs. The relationship between paternal coffee consumption and offspring NDs was also assessed, as a negative control (i.e. if the negative effects of coffee consumption are mediated by maternal/intrauterine effects then similar associations should not be seen in fathers). Offspring birth year and parental ages at birth were included as covariates in both analyses. All linear regression analyses were conducted using R version 4.1.1.

We ran additional linear regression analyses where we also included additional potential confounders. These were maternal and paternal education, maternal and paternal income, maternal smoking before pregnancy, at week 15 and week 30, maternal alcohol consumption before pregnancy, at week 12, week 15, week 24, and week 30. By including smoking and alcohol related variables, this model attempts to remove any direct confounding effect of those variables as well as the potential confounding effect of maternal impulsivity (i.e. genetic confounding) (Havdahl et al., 2022). We also included binary (yes/no) variables for maternal smoking and alcohol consumption at each time point.

In a separate model, we attempted to isolate the effect of maternal coffee consumption during pregnancy (i.e. the intrauterine effect) by controlling for maternal coffee consumption before pregnancy. This model included the covariates maternal coffee consumption before pregnancy, offspring birth year, maternal age at birth, paternal age at birth, maternal smoking before pregnancy, at week 15, and week 30, maternal alcohol before pregnancy, at week 15, week 24, and week 30, maternal and paternal income, maternal and paternal education, and offspring genotyping batch. We also included binary variables for maternal smoking and alcohol consumption at each timepoint.

Similar analyses were conducted in the genotyped subset of MoBa using a genetic linear mixed model approach that is more robust to the effects of cryptic relatedness and population structure (see online Supplementary Materials 2).

### Mendelian randomization analyses

Individual-level and Two-Sample MR analyses were performed to assess the causal nature of the relationship between maternal coffee consumption during pregnancy and offspring NDs. Power calculations for these analyses are presented in online Supplementary Materials 3.

#### Genetic variant selection

Eight independent genome-wide significant genetic variants that have been previously associated with coffee consumption (Cornelis et al., 2015) (log10Bayes-factor>5.64, ST2) were aggregated into weighted and unweighted polygenic scores (PGS) to be used in the MR analyses.

#### Benchmarking checks

We assessed whether the coffee PGS and individual SNPs were associated with maternal coffee consumption before and during pregnancy in MoBa while adjusting for offspring birth year, parental ages at birth, and maternal genotyping batch using a genetic linear mixed model. A maternal GRM was included in the random effects part of the model.

#### Individual-Level MR analyses

In the present study, we used genetic association analyses to inform on potential causal relationships, and these analyses are hereafter referred to as ‘individual-level MR analyses’. We assessed whether maternal weighted and unweighted PGS for coffee consumption were associated with offspring NDs using a genetic linear mixed model. This approach was chosen to account for cryptic relatedness and genetic confounding (see Discussion). The model incorporated an offspring GRM from all genotyped and imputed autosomal loci, excluding the eight coffee variants and 1 MB either side of these variants (to avoid modelling the same signal in both the fixed and random effect part of the model). The unweighted PGS analyses included terms for offspring unweighted PGS, offspring birth year, parental ages at birth, and both offspring and maternal genotyping batches. The offspring unweighted PGS was included as a covariate to block potential pleiotropic pathways through the offspring genome (D'Urso et al., 2021; Lawlor et al., 2017). The weighted PGS analyses conditioned upon offspring genotype (at all loci) rather than the PGS, to fully block potential pleiotropic pathways through the offspring genome.

We ran MR sensitivity analyses in the smaller subset of parent-offspring trios. These analyses were conducted to investigate the potential collider pathway introduced in the mother-child duo analyses when conditioning on offspring genome, but not the paternal genome, if a postnatal (paternal) pathway from paternal genotype to offspring NDs were present. Here, we assessed whether maternal weighted (or unweighted) PGS for coffee consumption were associated with offspring NDs using a genetic linear mixed model, while adjusting for offspring genotype (or unweighted PGS), paternal genotype (or unweighted PGS), offspring birth year, parental ages at birth, and all genotyping batches. We also estimated the effect of the paternal PGS conditional on offspring genome, maternal genome, offspring birth year, parental ages at birth, and all genotyping batches. This analysis served as a negative control, allowing us to evaluate the presence of a postnatal (confounding) effect of coffee consumption on offspring NDs.

#### Two-sample MR analyses

The Inverse Variance Weighted (IVW) Two-Sample MR approach was applied to investigate the relationship between maternal coffee consumption and offspring NDs (Burgess, Butterworth, & Thompson, 2013). SNP-exposure data were extracted from the external GWAS of coffee consumption (Cornelis et al., 2015) (ST2).

SNP-outcome data for the Two-Sample MR analyses (as well as the multivariable MR analyses; see below) came from our own analyses within the MoBa cohort. For analyses of maternal exposures on offspring outcomes, we require estimates of the effect of the maternal genotype on offspring outcome, with the effect of the offspring genotype removed. Therefore, maternal and offspring GWAS of the ND outcomes were conducted and the genetic effects were then partitioned to provide estimates of the maternal-SNP-outcome effect. The GWAS were implemented using the fastGWA linear mixed model approach (Jiang et al., 2019). A GRM of all mothers and offspring was generated and converted into a sparse GRM before being included in the maternal and offspring GWAS. We included offspring birth year, parental age at birth, and ten offspring and maternal genetic PCs as covariates. The maternal GWAS of NDs were limited to one pregnancy per mother (first-born child in MoBa). The maternal and offspring genetic effects on each ND were partitioned using the Direct and INdirect effects analysis of Genetic lOci (DINGO) approach (Hwang et al., 2023). The effective sample overlap between the offspring and maternal GWAS for DINGO was estimated using bivariate linkage disequilibrium (LD) score regression and the 1000 Genomes Project European reference panel (Bulik-Sullivan et al., 2015a, 2015b) (see ST3 for GWAS sample sizes and bivariate LD score regression estimates used for partitioning the genetic effects).

Two Sample MR analyses were performed using the TwoSampleMR package (Hemani et al., 2018) (https://github.com/MRCIEU/TwoSampleMR) in R version 4.1.1 (https://cran.r-project.org/). Sensitivity analyses were performed using the MR-Egger (Bowden, Davey Smith, & Burgess, 2015), Weighted Median (Bowden, Davey Smith, Haycock, & Burgess, 2016), Simple Mode and Weighted Mode (Hartwig, Davey Smith, & Bowden, 2017) methodologies. Heterogeneity tests of the causal effect estimates were conducted using Cochran's Q. Directional pleiotropy was assessed through the MR–Egger intercept. I^2^_GX_ was calculated to evaluate the risk for weak instrument bias in the MR-Egger analyses. Exposure and outcome GWAS summary statistics are provided in ST4.

#### Investigations into pleiotropy

Several additional sensitivity analyses were performed to investigate potential pleiotropic effects of the SNPs and PGS used in the MR analyses (i.e. violations of MR assumptions). Firstly, we examined whether coffee SNPs and PGS were associated with maternal alcohol consumption (amongst consumers), smoking (amongst smokers), education and income, as well as binary (yes/no) variables for smoking and alcohol consumption. These analyses were performed using GCTA and adjusted for offspring birth year, parental ages at birth and maternal genotyping batch. If associations were identified, we performed additional MR analyses to control for the potential confounders. Firstly, additional individual-level MR analyses were repeated in mother-child duos using weighted PGS, this time adjusting for variables that the coffee PGS were associated with (as well as offspring genome, offspring birth year, parental ages at birth and genotyping batches). Secondly, multivariable IVW MR (MVMR) was employed to estimate the direct causal effect of maternal coffee exposure on offspring NDs, conditional on smoking heaviness and alcohol frequency. Educational attainment was not included in the MVMR (see Discussion). Genetic instrumental variables for smoking heaviness (cigarettes/day) and alcohol frequency (weekly) were identified in the ancestry-stratified GWAS meta-analyses of Europeans (excluding 23&Me) (Saunders et al., 2022). Summary statistics were clumped (*p* < 5 × 10^−8^, r^2^ = 0.001), and palindromic SNPs were removed, resulting in 17 and 48 SNPs for smoking and alcohol frequency (that were available across all exposure and outcome GWAS data) respectively. Coffee consumption was proxied by the aforementioned genetic variants. The maternal genetic effects on offspring ND outcomes were used for the outcome data. MVMR analyses were performed using the TwoSampleMR package and the input summary statistics are provided in ST5.

#### Gene by environment interaction MR

The GxE MR framework (Chen et al., 2008; Davey Smith, 2010) was also utilized as a sensitivity analysis, where two additional individual-level MR analyses were performed in subgroups stratified by coffee consumption status during pregnancy. Consumers were defined as women who had reported consuming coffee in the week 15 or week 22 Questionnaire (*N* = 35 250 mothers), while never consumers were defined as those who reported no coffee consumption across both timepoints (*N* = 8544 mothers). In order to investigate the possibility of introducing collider bias to these analyses when conditioning on coffee consumption (see Discussion), genetic variants were tested for association with ever/never consuming coffee during pregnancy using a genetic linear mixed model in GCTA (*N* = 43 794). This model adjusted for offspring birth year, maternal age at birth, and maternal genotyping batch, and included a GRM. Any variants significantly associated with ever/never consuming coffee were excluded from the weighted PGS used in the GxE MR. The GxE MR analyses assessed whether maternal weighted PGS for coffee consumption were associated with offspring NDs (amongst ever consumers and never consumers) using the previously described genetic linear mixed model (i.e. while adjusting for offspring genotype, offspring birth year, parental ages at birth, and maternal and offspring genotyping batches) and included a GRM. A heterogeneity test was conducted to compare results from the consumer and non-consumer stratified analyses.

## Results

Descriptive characteristics of the MoBa cohort after QC are presented in Table 1. Statistical power to detect causal effect estimates in the present study is shown in online Supplementary Materials 3.

**Table 1.** Characteristics of the MoBa mothers, fathers and offspring after quality control, prior to neurodevelopmental difficulty (ND; age assessed) rank-based inverse normal transformation. Summary statistics were calculated amongst consumers for the coffee, smoking and alcohol related variables

*Birth characteristics*	Mean	s.d.	Minimum	Maximum	N
Offspring birth year	2005	2	1999	2009	71 443
Offspring sex (% male)	51				71 443
Maternal age at birth (years)	30	5	16	46	71 439
Paternal age at birth (years)	33	5	18	61	71 296
*Offspring NDs*	Mean	s.d.	Minimum	Maximum	N
*Difficulties with social communication and behavioral flexibility* (*repetitive behaviors*)					
SCQ-full (3 years)	6.2	3.3	0	31	40 046
SCQ-full (8 years)	3.4	2.9	0	36	29 458
SCQ-RRB (3 years)	3.8	2.5	0	12	39 974
SCQ-RRB (8 years)	0.6	1.1	0	12	29 599
SCQ-SCI (3 years)	2.3	1.8	0	23	40 044
SCQ-SCI (8 years)	2.6	2.4	0	25	29 442
*Difficulties with attention and hyperactive-impulsive behavior*					
CBCL-ADHD (18 months)	2.6	1.6	0	8	48 172
CBCL-ADHD (3 years)	3.4	2.2	0	12	39 964
CBCL-ADHD (5 years)	2.5	2.2	0	12	28 020
RS-DBD-ADHD (8 years)	8.4	7.1	0	54	29 580
RS-DBD-INA (8 years)	4.9	4.1	0	27	29 579
RS-DBD-HYP (8 years)	3.5	3.9	0	27	29 571
CPRS (5 years)	4.3	4.5	0	36	28 026
*Language difficulties*					
ASQ-LANG (18 months)	1.2	1.5	0	6	51 804
ASQ-LANG (3 years)	0.6	1.1	0	12	40 081
ASQ-LANG (5 years)	0.7	1.2	0	14	27 946
CCC-S (8 years)	4.6	4.2	0	37	29 497
*Motor difficulties*					
ASQ-MOTOR (18 months)	0.7	1.3	0	12	51 868
ASQ-MOTOR (3 years)	1.2	1.3	0	8	39 952
CDI-MOTOR (5 years)	0.8	1.4	0	12	28 011
*Parental consumption characteristics*	Mean	s.d.	Range	% Consumers	Total N
Maternal Coffee (cups/day; before pregnancy)	2.4	2.5	1–35	55	37 611
Maternal Coffee (cups/day; week 15)	1.6	1.1	1–35	43	44 597
Maternal Coffee (cups/day; week 22)	1.2	1.3	0.03–24.5*	65	56 708
Maternal Smoking (cigarettes/week; before)	53.8	47.7	1–420	31	58 600
Maternal Smoking (cigarettes/week; week 15)	36	30.4	1–245	9	59 579
Maternal Smoking (cigarettes/week; week 30)	37.7	31	1–280	7	63 392
Maternal Alcohol Frequency (weekly; before)	0.7	0.8	0.1–6.5**	93	63 358
Maternal Alcohol Frequency (weekly; week 12)	0.3	0.5	0.1–6.5**	28	57 480
Maternal Alcohol Frequency (weekly; week 15)	0.2	0.4	0.1–6.5**	13	57 650
Maternal Alcohol Frequency (weekly; week 24)	0.3	0.4	0.1–6.5**	11	55 758
Maternal Alcohol Frequency (weekly; week 30)	0.3	0.4	0.1–6.5**	11	55 846
Paternal Coffee (cups/day)	2.9	1.5	0.4–12*	82	21 691
Maternal Caffeine (mg/day; before pregnancy)	71.0	104.9	2–992	89	48 582
Maternal Caffeine (mg/day; week 15)	46.6	52.9	2–960	82	47 328
Maternal Caffeine (mg/day; week 22)	54.2	67.08	0.07–820.5	98	56 735
Paternal Caffeine (mg/day)	129.03	82.7	5.14–649.5	97	21 177
*Socioeconomic variables*	Mean	s.d.	Minimum	Maximum	Total N
Maternal gross yearly income (NOK)	259 065.5	120 585.08	0	500 000	65 363
Paternal gross yearly income (NOK)	344 566.5	117 492.3	0	500 000	63 154
Maternal education (years)	14.1	2.4	9	17	64 278
Paternal education (years)	13.5	2.7	9	17	61 810

N, Sample size; s.d., Standard deviation; NOK, Norwegian Kroner; SCQ-full, Social Communication Questionnaire; SCQ-RRB, Social Communication Questionnaire restricted and repetitive behavior subscale; SCQ-SCI, Social Communication Questionnaire social communication impairment subscale; CBCL-ADHD, Child Behavior Checklist ADHD subscale; RS-DBD-ADHD: Rating Scale for Disruptive Behavior Disorders ADHD subscale; RS-DBD-INA, Rating Scale for Disruptive Behavior Disorders inattention subscale; RS-DBD-HYP, Rating Scale for Disruptive Behavior Disorders hyperactivity subscale; CPRS: Conners Parent Rating Scale-Revised short form; ASQ-LANG, Ages and Stages Questionnaire language subscale; CCC-S, The Children's Communication Checklist-2 Short Scale; ASQ-MOTOR, Ages and Stages Questionnaire motor items; CDI-MOTOR, Child Development Inventory motor subscale.

*non-whole number due to converting weekly consumption to daily.

**non-whole number due to converting monthly consumption to weekly.

### Traditional epidemiological analyses

**Figure 1.**
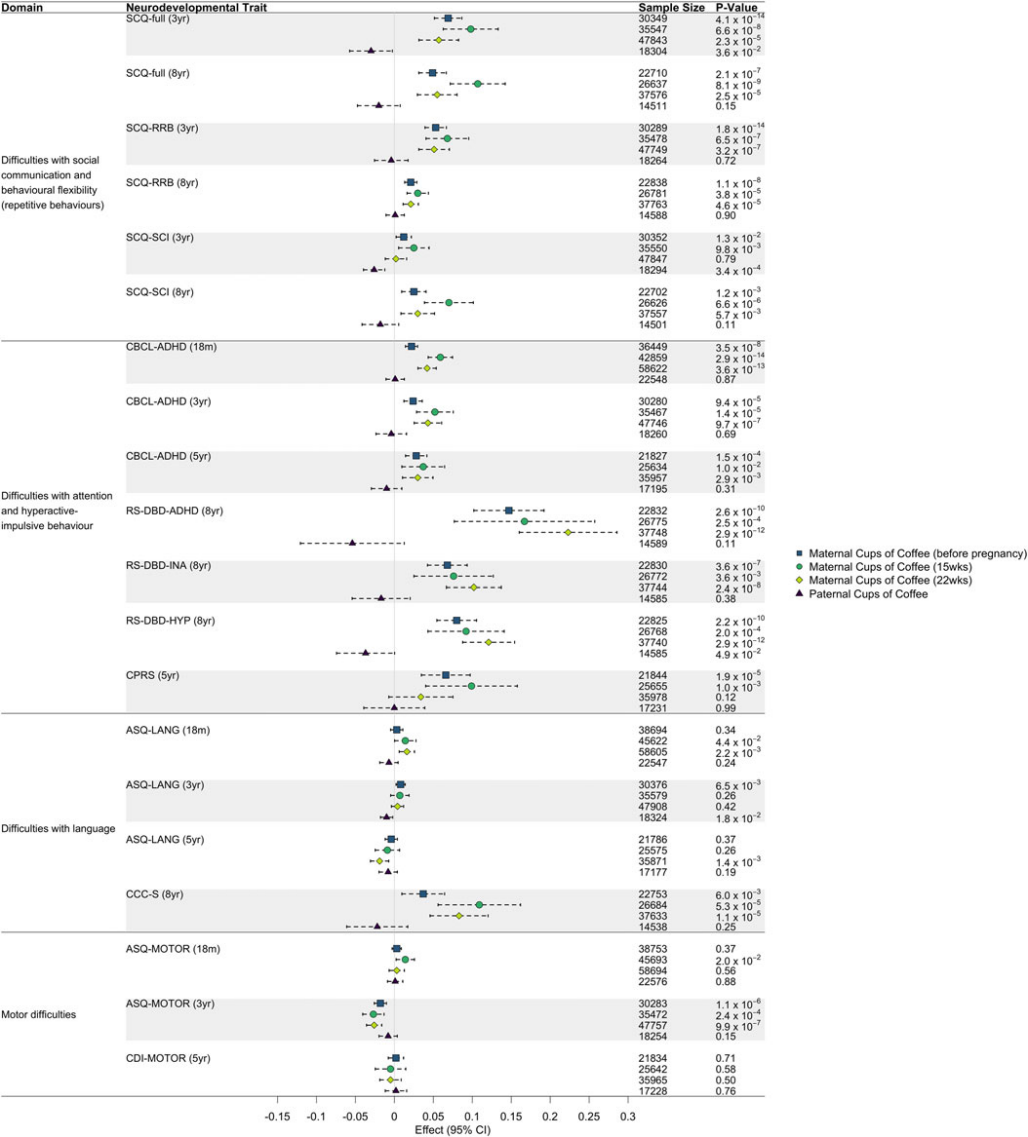
Effect estimates and 95% confidence intervals (CI) from the traditional observational analyses assessing the relationship between both maternal and paternal coffee consumption exposure (cups/day) and offspring neurodevelopmental difficulties (ND) outcomes (rank-based inverse normal transformed) using linear regression. Covariates included offspring birth year, maternal age at birth and paternal age at birth. SCQ-full, Social Communication Questionnaire; SCQ-RRB, Social Communication Questionnaire restricted and repetitive behavior subscale; SCQ-SCI: Social Communication Questionnaire social communication impairment subscale; CBCL-ADHD: Child Behavior Checklist ADHD subscale; RS-DBD-ADHD: Rating Scale for Disruptive Behavior Disorders ADHD subscale; RS-DBD-INA: Rating Scale for Disruptive Behavior Disorders inattention subscale; RS-DBD-HYP: Rating Scale for Disruptive Behavior Disorders hyperactivity subscale; CPRS, Conners Parent Rating Scale-Revised short form; ASQ-LANG: Ages and Stages Questionnaire language subscale; CCC-S: The Children's Communication Checklist-2 Short Scale; ASQ-MOTOR: Ages and Stages Questionnaire motor items; CDI-MOTOR: Child Development Inventory motor subscale.

The unadjusted traditional observational analyses (i.e. not adjusting for smoking/alcohol/education/income) found strong evidence for positive associations between maternal coffee consumption and several measures related to offspring difficulties with social communication and behavioral flexibility (Fig. 1; *p* < 0.005). Likewise, there was strong evidence of positive associations between maternal coffee consumption and all offspring difficulties with attention and hyperactive-impulsive behavior (Fig. 1; *p* < 0.005). There was also some evidence for positive associations between maternal coffee consumption and language difficulties at ages 18 months, 3 years, and 8 years (Fig. 1; *p* < 0.005). Interestingly, negative associations were observed between maternal coffee consumption and language difficulties at age 5 and motor difficulties at age 3 (Fig. 1; *p* < 0.005). There was little evidence that paternal coffee consumption was associated with the NDs, apart from social communication difficulties at age 3 years (*p* < 0.005; negative association).

The results from the observational analyses that adjusted for education, income, smoking and alcohol related variables can be found in online Supplementary Materials 4. Many of the previously significant effects attenuated towards zero and some associations switched from being positive to negative. Significant effects only remained for social communication and behavioral flexibility difficulties at age 3, difficulties with attention and hyperactive-impulsive behavior at age 5, and motor difficulties at age 3 (*p* < 0.005).

When adjusting for maternal coffee consumption before pregnancy, we observed significant negative associations between maternal coffee consumption during pregnancy and offspring difficulties with social communication and behavioral flexibility at age 3, difficulties with attention and hyperactive-impulsive behavior at age 5, and motor difficulties at age 3 (online Supplementary Materials 5; *p* < 0.005).

Online Supplementary Materials 2 shows similar results for the observational analyses conducted in the genotyped MoBa sample.

### Mendelian randomization analyses

#### Benchmarking checks

Benchmarking analyses found that both the weighted and unweighted maternal PGS for coffee consumption were positively associated with self-reported maternal coffee consumption in MoBa across all timepoints (ST6; *p* < 0.05). As for the individual SNPs, all except for rs1481012 (*ABCG2*), showed evidence for positive association with coffee consumption for at least one time point in MoBa (ST7; *p* < 0.05). Likewise, the I^2^_GX_ and F statistics for individual SNPs and PGS are given in ST8 and ST9, respectively (all F > 10 and I^2^_GX_  > 0.9). Together, this suggests that the GRS are appropriate genetic instrumental variables for maternal coffee consumption during pregnancy and that weak instrument bias is unlikely to be an issue.

#### Individual-Level MR analyses

The mother-child duo individual-level MR analyses found some evidence for association between PGS for increased coffee intake and offspring NDs (Table 2). The weighted PGS was positively associated with social communication difficulties at age 8 years (beta = 0.128; se = 0.043, *p* = 0.003), although there was little evidence for association between the unweighted PGS and any of the outcomes (Table 2).

**Table 2.** Results from weighted and unweighted polygenic score (PGS) analyses in mother-child duos. A genetic linear mixed model was used to assess the relationship between maternal PGS and offspring neurodevelopmental difficulties (NDs). Weighted PGS were calculated as the summed dosage of each SNP weighted by the effect size (cups/day), whereas unweighted PGS are the summed dosage of coffee consumption increasing alleles. NDs were rank-based inverse normal transformed. Covariates for the weighted PGS analyses included offspring genotypes, offspring birth year, maternal age at birth, paternal age at birth and both maternal and offspring genotyping batch, whereas the unweighted PGS analysis covariates include offspring unweighted PGS (as opposed to genotypes). The effect estimates can be interpreted as the expected increase in outcome per unit change in the PGS

			Maternal Weighted PGS			Maternal Unweighted PGS		
Domain	ND	N	Beta	SE	P	Beta	SE	P
Difficulties with social communication and behavioral flexibility (repetitive behaviors)	**SCQ-full (3 years)**	35 687	−0.217	0.131	0.097	−0.025	0.011	**0.031**
	**SCQ-full (8 years)**	26 156	0.106	0.044	**0.016**	0.005	0.004	0.177
	**SCQ-RRB (3 years)**	35 619	−0.078	0.038	**0.041**	−0.009	0.003	**0.008**
	SCQ-RRB (8 years)	26 285	0.001	0.036	0.986	−0.002	0.003	0.599
	SCQ-SCI (3 years)	35 686	−0.007	0.037	0.852	0.000	0.003	0.948
	**SCQ-SCI (8 years)**	26 141	0.128	0.043	0.003*	0.008	0.004	**0.042**
Difficulties with attention and hyperactive-impulsive behavior	CBCL-ADHD (18 months)	42 944	−0.061	0.034	0.075	−0.004	0.003	0.146
	CBCL-ADHD (3 years)	35 618	−0.041	0.038	0.291	−0.004	0.003	0.238
	CBCL-ADHD (5 years)	24 955	0.003	0.044	0.943	−0.004	0.004	0.326
	RS-DBD-ADHD (8 years)	26 268	−0.010	0.044	0.813	−0.002	0.004	0.587
	RS-DBD-INA (8 years)	26 267	−0.003	0.044	0.941	0.000	0.004	0.961
	RS-DBD-HYP (8 years)	26 259	−0.016	0.042	0.711	−0.004	0.004	0.337
	CPRS (5 years)	24 960	−0.040	0.044	0.361	−0.004	0.004	0.284
Language difficulties	**ASQ-LANG (18 months)**	46 188	0.037	0.029	0.197	−0.005	0.003	**0.033**
	ASQ-LANG (3 years)	35 716	0.008	0.031	0.810	0.001	0.003	0.844
	ASQ-LANG (5 years)	24 887	−0.002	0.038	0.960	0.002	0.003	0.558
	CCC-S (8 years)	26 188	0.021	0.044	0.642	0.000	0.004	0.985
Motor difficulties	ASQ-MOTOR (18 months)	46 245	0.006	0.027	0.828	−0.002	0.002	0.513
	ASQ-MOTOR (3 years)	35 602	−0.063	0.033	0.059	−0.003	0.003	0.357
	CDI-MOTOR (5 years)	24 948	−0.028	0.037	0.456	0.001	0.003	0.829

*p* < 0.05 = bold, *p* < 0.005 = *

PGS, polygenic score; SE, standard error; *p*, P-value; SCQ-full, Social Communication Questionnaire; SCQ-RRB, Social Communication Questionnaire restricted and repetitive behavior subscale; SCQ-SCI, Social Communication Questionnaire social communication impairment subscale; CBCL-ADHD, Child Behavior Checklist ADHD subscale; RS-DBD-ADHD, Rating Scale for Disruptive Behavior Disorders ADHD subscale; RS-DBD-INA, Rating Scale for Disruptive Behavior Disorders inattention subscale; RS-DBD-HYP, Rating Scale for Disruptive Behavior Disorders hyperactivity subscale; CPRS, Conners Parent Rating Scale-Revised short form; ASQ-LANG, Ages and Stages Questionnaire language subscale; CCC-S, The Children's Communication Checklist-2 Short Scale; ASQ-MOTOR, Ages and Stages Questionnaire motor items; CDI-MOTOR, Child Development Inventory motor subscale.

The parent-child trio analyses (ST10) found some evidence that maternal PGS were associated with difficulties with attention and hyperactive-impulsive behavior (beta = −0.131, se = 0.044, *p* = 0.003) and language difficulties at 18 months (beta = −0.009, se = 0.003, *p* = 0.004). In contrast, there was little evidence for association between paternal PGS and offspring NDs in the negative control analyses in parent-child trios (ST11; all *p* > 0.005).

#### Two-sample MR analyses

The IVW MR analyses found a suggestive positive association (Table 3; *p* < 0.05) between coffee consumption and social communication difficulties at age 8 (beta = 0.348, se = 0.141; *p* = 0.014).

**Table 3.** Two sample Inverse Variance Weighted Mendelian randomization results. Shown are the causal effect estimates (beta) from analyses of maternal coffee exposure (proxied by 8 SNPs associated with coffee consumption) on offspring neurodevelopmental difficulties (NDs). NDs were rank-based inverse normal transformed. The units for the causal effects are per unit increase in the outcome per extra cup of coffee consumed per day

Domain	ND	Beta	SE	P
Difficulties with social communication and behavioral flexibility (repetitive behaviors)	SCQ-full (3 years)	−0.155	0.144	0.282
	SCQ-full (8 years)	0.264	0.156	0.090
	SCQ-RRB (3 years)	−0.180	0.116	0.119
	SCQ-RRB (8 years)	−0.070	0.059	0.240
	SCQ-SCI (3 years)	0.011	0.079	0.890
	**SCQ-SCI (8 years)**	0.348	0.141	**0.014**
Difficulties with attention and hyperactive-impulsive behavior	CBCL-ADHD (18 months)	−0.126	0.066	0.057
	CBCL-ADHD (3 years)	−0.092	0.136	0.502
	CBCL-ADHD (5 years)	−0.011	0.118	0.928
	RS-DBD-ADHD (8 years)	0.265	0.372	0.477
	RS-DBD-INA (8 years)	0.095	0.211	0.652
	RS-DBD-HYP (8 years)	0.190	0.214	0.374
	CPRS (5 years)	−0.329	0.250	0.188
Difficulties with language	ASQ-LANG (18 months)	−0.089	0.095	0.351
	ASQ-LANG (3 years)	0.011	0.048	0.820
	ASQ-LANG (5 years)	0.014	0.078	0.857
	CCC-S (8 years)	0.051	0.227	0.823
Motor difficulties	ASQ-MOTOR (18 months)	−0.022	0.053	0.671
	ASQ-MOTOR (3 years)	−0.103	0.058	0.075
	CDI-MOTOR (5 years)	0.021	0.076	0.786

*p* < 0.05 bolded

SE, standard error; *p*, *P*-value; SCQ-full, Social Communication Questionnaire; SCQ-RRB, Social Communication Questionnaire restricted and repetitive behavior subscale; SCQ-SCI, Social Communication Questionnaire social communication impairment subscale; CBCL-ADHD, Child Behavior Checklist ADHD subscale; RS-DBD-ADHD, Rating Scale for Disruptive Behavior Disorders ADHD subscale; RS-DBD-INA, Rating Scale for Disruptive Behavior Disorders inattention subscale; RS-DBD-HYP, Rating Scale for Disruptive Behavior Disorders hyperactivity subscale; CPRS, Conners Parent Rating Scale-Revised short form; ASQ-LANG, Ages and Stages Questionnaire language subscale; CCC-S, The Children's Communication Checklist-2 Short Scale; ASQ-MOTOR, Ages and Stages Questionnaire motor items; CDI-MOTOR, Child Development Inventory motor subscale.

### Sensitivity analyses

To avoid over-interpretation of results, sensitivity analyses were focused upon NDs where there was some evidence (*p* < 0.005) of association in the main individual-level MR and two-sample MR analyses (i.e. social communication difficulties at age 8 years; SCI-SCI-8 yr).

#### Two-sample MR sensitivity analyses

The Weighted Median and Weighted Mode MR analyses all showed evidence for a positive causal effect between maternal coffee consumption and offspring social communication difficulties at age 8 years (weighted median: beta 0.366, se = 0.152, *p* = 0.016; weighted mode: beta = 0.386, se = 0.153, *p* = 0.040), while the MR-Egger and Simple Mode did not (ST12; Egger: beta = 0. 510, se = 0.300 *p* = 0.140; simple mode: beta = 0.386, se = 0.238, *p* = 0.149). There was no evidence of significant directional pleiotropy or heterogeneity for MR analyses of social communication difficulties at age 8 years (*p* > 0.05; ST13).

#### Investigation into pleiotropy

The coffee consumption increaser allele of rs9902453 (G; *EFCAB5*), was found to be nominally associated with maternal smoking intensity (ST14; Week 15: beta = 1.543, se = 0.608, *p* = 0.011; Week 30: beta = 1.763, se = 0.689, *p* = 0.010) and rs1481012 (G; *ABCG2*), rs4410790 (C; *AHR*) and rs6265 (C; *BDNF*) were associated with binary smoking (respectively; OR = 0.994, se = , 9, *p* = 0.017; OR = 0.997, se = 0.002, *p* = 0.029; OR = 1.007, se = 0.004, *p* = 0.046). The increaser allele of rs4410790 (C; *AHR*) and rs2472297 (T; *CYP1A2*) were positively associated with maternal alcohol consumption (ST15; Week 30: beta = 0.020, se = 0.007, *p* = 0.007 and week 30: OR = 1.005, se = 0.002, *p* = 0.037), while interestingly the increaser allele of a number of SNPs showed a decreased odds of initiation (rs4410790, rs1260326 and rs9902453; *p* < 0.05 at multiple time points). We found the increaser allele of one SNP rs1260326 (C; *GCKR*) was negatively associated with education (ST16; beta = −0.029, se = 0.014, *p* = 0.035) and income (ST16; beta = −1462.350, se = 675.261, *p* = 0.030).

Nominally significant positive associations were observed between the weighted PGS and maternal alcohol consumption at week 30 (ST17; beta = 0.067, se = 0.032, *p* = 0.039). The unweighted PGS was positively associated with smoking at week 15 (ST18; beta = 0.563, se = 0.244, *p* = 0.021) and education (ST19; beta = 0.017, se = 0.009, *p* = 0.043), and a decreased risk of smoking (yes *v.* no) at week 30 (ST18; beta = −0.002, se = 0.001, *p* = 0.023) and alcohol consumption (yes *v.* no) (ST17; week 12: beta = −0.003, se = 0.001, *p* = 0.004; week 24: beta = −0.002, se = 0.001, *p* = 0.007; week 30: beta = −0.002, se = 0.001, *p* = 0.043). No PGS were associated with income (ST19).

The individual-level MR analyses that adjusted for potential confounding variables showing association with the PGS (smoking, alcohol and education), found the weighted coffee PGS no longer showed significant evidence for association with social communication difficulties at age 8 years (ST20; beta = 0.088, se = 0.049, *p* = 0.071). The MVMR analysis including coffee consumption, smoking and alcohol consumption, found coffee consumption was independently and positively associated with social communication difficulties at age 8 years (ST21; beta = 0.37, se = 0.13, *p* = 0.004).

#### Gene by environment interaction MR

One genetic variant (rs78000944; *MLXIPL*) was significantly associated with ever/never consuming coffee during pregnancy (ST22; OR = 1.009, se = 0.003, *p* = 0.002), and was therefore excluded from the PGS used in the GxE MR. The GxE MR analyses found weak evidence for a positive association between coffee consumption and social communication difficulties at age 8 years amongst mothers who consumed coffee during pregnancy (beta = 0.153, se = 0.071, *p* = 0.032, *N* = 16 471). Although not statistically significant, we also detected a positive effect of similar magnitude amongst mothers who did not consume coffee during pregnancy (beta = 0.107, se = 0.134, *p* = 0.424, *N* = 4304). There was no evidence of heterogeneity in the consumer and non-consumer GxE MR effect estimates (*p* = 0.619).

## Discussion

This study utilized data from a large cohort of genotyped parent-offspring trios to investigate the relationship between maternal coffee consumption and offspring childhood NDs. Our observational analyses, which did not adjust for potential confounders, found that increased maternal coffee consumption was associated with many offspring NDs. However, after adjusting for potential confounders (maternal smoking, maternal alcohol consumption, parental education, and parental income), the previously significant effects of maternal coffee consumption on offspring ND outcomes attenuated towards zero. Our findings agree with previous observational work conducted within an earlier, but much smaller version of MoBa (version 8 released in 2015; Berglundh et al., 2021), which included similar outcomes (excluding the Social Communication Questionnaire and the Conners Parent Rating Scale) and covariates.

Our MR analyses found little evidence for a causal effect between maternal coffee consumption and most offspring NDs. Although we observed a significant positive association with social communication difficulties at age 8 years, we were unable to confidently rule out the possibility of pleiotropic pathways driving this association. Importantly, the proposed mechanisms of action for SNPs associated with coffee consumption is highly heterogeneous, with possible roles spanning caffeine metabolism, reward response, taste perception, and additional unknown processes, and may therefore invalidate one of the core MR assumptions (i.e. exclusion restriction assumption) (Brito Nunes et al., 2022; Cornelis & Munafo, 2018). Although we did not detect pleiotropy in our MR sensitivity analyses, we took precautions to address this potential issue. Since many of the SNPs/PGS showed evidence for association with potential confounders (smoking/alcohol/education) in MoBa, we conducted additional individual-level MR analyses, that adjusted for these variables. Here, we found that the effect between coffee consumption and social communication difficulties at age 8 years weakened and was no longer significant.

We also attempted to genetically proxy potential confounders. The MVMR analyses found that coffee consumption was significantly associated with social communication difficulties at age 8 years, independent of smoking and alcohol consumption. However, we omitted educational attainment from the MVMR analyses, to avoid potential bias in the MR results stemming from using variants known to be subject to population stratification (Haworth et al., 2019), assortative mating (Domingue, Fletcher, Conley, & Boardman, 2014; Howe, Nivard, et al., 2022; Robinson et al., 2017; Yengo et al., 2018), and involve indirect genetic effects (Howe, Evans, Hemani, Davey Smith, & Davies, 2022; Howe, Nivard, et al., 2022; Kong et al., 2018; Lee et al., 2018; Wang et al., 2021), rather than direct genetic effects (Howe et al., 2023). While these findings provide some evidence for a causal effect of coffee consumption, it is possible that education (whose association with child ADHD symptoms persists after controlling for shared genetic and family environmental factors (Torvik et al., 2020)) or some other non-modelled confounding variable, could be driving the association.

In presence of a true causal effect of maternal coffee consumption on offspring NDs, we expected our GxE MR analyses to detect an effect only amongst coffee consumers, and not in the non-consumers. Our analyses found a causal effect of similar magnitude in both the consumers and non-consumers, suggesting that pleiotropy may be driving the association between maternal coffee consumption and social communication difficulties at age 8 years. It is worth noting that the proportion of mothers abstaining from coffee within the MoBa dataset is small, consistent with Norway's high coffee consumption rates (International Coffee Council, 2012; Lukic et al., 2020), leading to larger standard errors and reduced statistical power in the ‘never consuming’ analysis, possibly explaining why the effect (of similar magnitude) was non-significant in this group. Furthermore, coffee consumption may be a collider (Cornelis & Munafo, 2018) and conditioning upon consumption in the GxE MR analyses may have introduced a path between the genetic instrumental variables and confounders of the exposure outcome relationship (online Supplementary Materials 6). We attempted to minimize this effect by excluding SNPs associated with ever/never consuming coffee during pregnancy in MoBa, however it is possible that the remaining variants could also be associated with ever/never consuming coffee. In addition, it is possible that mothers who drink coffee may be systematically different from mothers who don't in terms of how they report symptoms in their children, and the GxE MR framework is not robust to this.

Unlike traditional observational approaches, our within-family MR is robust to some forms of genetic confounding. For example, if a SNP relates to maternal coffee consumption, maternal ADHD and offspring NDs, the effect of an offspring inheriting that SNP is accounted for through inclusion of offspring genotype in the same model (i.e. essentially blocking the path from maternal to offspring genome). Indeed, a study has shown that pregnant individuals with high ADHD genetic liability are at increased risk of adverse pregnancy-related exposures (such as smoking during pregnancy), which have been linked to offspring neurodevelopmental outcomes (Havdahl et al., 2022), necessitating genetically informative methods to disentangle the complicated relationships between variables.

Importantly, we note that the weightings used in the MR analyses were derived from a GWAS of non-pregnant individuals, and consequently may not accurately reflect effect sizes in pregnant women. Our benchmarking analyses found that the SNPs and PGS were generally positively associated with coffee consumption during pregnancy in MoBa. This mirrors results from a previous study that found the same PGS were positively associated with coffee consumption at week 32 of gestation in the ALSPAC cohort (Brito Nunes et al., 2022). While this shows our instruments can proxy coffee consumption during pregnancy, the weightings may not be accurate. Therefore, rather than focusing on the magnitude of the causal effect estimates, we suggest that the presence/absence and direction of the effect may be more informative.

Notably, our MR analyses had limited statistical power, due to the PGS explaining minimal variance in coffee consumption. We attempted to increase statistical power by including six additional SNPs previously associated with coffee consumption in a large US/UK GWAS (Zhong et al., 2019) (*N* = 672 357), however these SNPs were not strongly or consistently associated with coffee consumption in MoBa (ST23). This difference may be due to differences in study populations (UK *v.* Norwegian), who may differ in their coffee consumption behavior (i.e. Norwegian preference to coffee, and a UK preference for tea). Therefore, these SNPs may not accurately capture the effect of coffee consumption in MoBa, rendering them unsuitable for this study.

There are several future study directions. This study focused on the effect of maternal coffee consumption on offspring difficulties with language, motor skills, social communication, behavioral flexibility, attention, and hyperactivity. The NDs were reported by the mothers and therefore may be subject to certain reporting biases. Consequently, it would be beneficial if future studies investigated clinical assessments, cognitive tests, and teacher ratings of offspring neurodevelopmental traits. Likewise, the effects of maternal coffee consumption on offspring neurodevelopmental disorder diagnoses should also be examined. Many other aspects of coffee consumption are also worth exploring. Our study did not explore whether the effects of maternal coffee consumption act during critical-periods, and we did not directly investigate caffeine, a likely mediator of a potential relationship between maternal coffee consumption and offspring outcomes. However, we observed strong positive associations between maternal (coffee) PGS/SNPs and caffeine intake (ST6 and ST7), suggesting that caffeine may also be proxied by the instruments used in the present study. Investigating the impact of various caffeine sources, such as soft drink and tea, on offspring outcomes is also relevant, as past observational studies have linked maternal soft drink consumption, rather than tea or coffee/caffeine, to childhood NDs (Bekkhus et al., 2010; Berglundh et al., 2021). We did not investigate dose-dependent effects or the influence of particular caffeine metabolites (i.e. theobromine, theophylline, paraxanthine). This may be relevant, as a previous observational study examining maternal serum paraxanthine concentrations and childhood cognitive and behavioral outcomes (at ages 4 and 7 years) found little evidence of an adverse association for the majority of pregnant women consuming moderate amounts of caffeine (Klebanoff & Keim, 2015). Importantly, coffee contains many bioactive compounds other than caffeine (e.g. chlorogenic acid, trigonelline, cafestol, kahweol) (Socała, Szopa, Serefko, Poleszak, & Wlaź, 2020; Spiller, 2019), which may impact neurodevelopment. As more genetic variants associated with caffeine consumption/metabolism become available, it may be possible to investigate these questions using MR. Lastly, our work was conducted in the MoBa cohort, which may not be representative of other populations, and similar analyses should be conducted in cohorts of different ancestries and ethnicities.

In conclusion, this study applied several conventional and genetic epidemiological approaches to investigate the potential relationship between maternal coffee consumption during pregnancy and offspring NDs. When considering the results of the conventional and genetic epidemiological analyses, and also the broader literature, we conclude that there is little evidence that maternal coffee consumption during pregnancy is strongly causally related to offspring NDs.

## Supporting information

D'Urso et al. supplementary material 1 D'Urso et al. supplementary material

D'Urso et al. supplementary material 2 D'Urso et al. supplementary material

## Data Availability

Data from the Norwegian Mother, Father and Child Cohort Study and the Medical Birth Registry of Norway used in this study are managed by the national health register holders in Norway (Norwegian Institute of Public Health) and can be made available to researchers, provided approval from the Regional Committees for Medical and Health Research Ethics (REC), compliance with the EU General Data Protection Regulation (GDPR) and approval from the data owners. The consent given by the participants does not open for storage of data on an individual level in repositories or journals. Researchers who want access to data sets for replication should apply through helsedata.no. Access to data sets requires approval from The Regional Committee for Medical and Health Research Ethics in Norway and an agreement with MoBa.

## Supplementary material

The supplementary material for this article can be found at https://doi.org/10.1017/S0033291724002216.
