# Supplementary material for: Mendelian randomization analysis of maternal coffee consumption during pregnancy on offspring neurodevelopmental difficulties in the Norwegian Mother, Father and Child Cohort Study (MoBa)
Source: Psychol Med. 2024 Oct 9;54(12):3548–61. doi: 10.1017/S0033291724002216 (PMC11496242; doi:10.1017/S0033291724002216)
Supplement: D'Urso et al. supplementary material 1 — D'Urso et al. supplementary material [file S0033291724002216sup001.docx]

Supplementary Materials

**Mendelian Randomization Analysis of Maternal Coffee Consumption During Pregnancy on Offspring Neurodevelopmental Difficulties in the Norwegian Mother, Father and Child Cohort Study (MoBa)**

Shannon D’Urso, BAdvSc(Hons)^1,11^, Robyn E Wootton, PhD^2,3,4^, Helga Ask, PhD^5,6^, Caroline Brito Nunes, BBiomedSc(Hons)^1^, Ole A Andreassen, MD, PhD^7^, Liang-Dar Hwang, PhD^1^, Gunn-Helen Moen, PhD^1,8,9,10^*, David M Evans, PhD^1,2,10^*, Alexandra Havdahl, PhD^2,4,5,6^*.

*Joint last authors

^1^Institute for Molecular Bioscience, The University of Queensland, Brisbane, Queensland, Australia

^2^MRC (Medical Research Council) Integrative Epidemiology Unit, University of Bristol, Bristol, United Kingdom

^3^School of Psychological Science, University of Bristol,  Bristol, UK

^4^Nic Waals Institute, Lovisenberg Diaconal Hospital, Oslo, Norway

^5^PsychGen Center for Genetic Epidemiology and Mental Health, Norwegian Institute of Public Health, Oslo, Norway

^6^PROMENTA Research Center, Department of Psychology, University of Oslo, Oslo, Norway

^7^NORMENT Centre, Division of Mental Health and Addiction, Institute of Clinical Medicine, University of Oslo, Oslo University Hospital, Oslo, Norway

^8^Institute of Clinical Medicine, Faculty of Medicine, University of Oslo, Oslo, Norway

^9^K. G. Jebsen Center for Genetic Epidemiology, Department of Public Health and Nursing, NTNU, Norwegian University of Science and Technology, Trondheim, Norway

^10^Frazer Institute, The University of Queensland, Woolloongabba, Queensland, Australia

^11^The University of Queensland

Corresponding author: Shannon D’Urso, BAdvSc, Institute for Molecular Bioscience, 306 Carmody Rd, St Lucia, QLD 4072, Australia ([s.durso@uq.edu.au](mailto:s.durso@uq.edu.au)).

Supplementary Contents

[Supplementary Materials 1. Descriptions of offspring neurodevelopmental difficulties 3](#_Toc156296566)

[Supplementary Materials 2. Principal Component Analysis of neurodevelopmental difficulties 5](#_Toc156296567)

[Supplementary Materials 3. Observational analyses using genetic linear mixed models. 6](#_Toc156296568)

[Supplementary Figure 1. Effect estimates and 95% confidence intervals (CI) from the observational analyses assessing the relationship between both maternal and paternal coffee consumption exposure (cups/day) and offspring neurodevelopmental difficulties (ND) outcomes (rank-based inverse normal transformed) using the linear mixed model approach. The linear mixed model was used to assess the relationship between maternal or paternal coffee consumption at each timepoint and offspring NDs, while adjusting for offspring birth year, maternal age at birth, paternal age at birth, and offspring genotyping batch. The model also included a genetic relationship matrix to model cryptic relatedness and population structure. 6](#_Toc156296569)

[Supplementary Figure 2. Effect estimates and 95% confidence intervals (CI) from the traditional observational analyses that adjusted for additional confounders assessing the relationship between both maternal and paternal coffee consumption exposure (cups/day) and offspring neurodevelopmental difficulties (ND) outcomes (rank-based inverse normal transformed). The linear mixed models adjusted for offspring birth year, maternal age at birth, paternal age at birth, maternal smoking before pregnancy, at week 15, and week 30, maternal alcohol before pregnancy, at week 15, week 24, and week 30, maternal and paternal income, maternal and paternal education, and offspring genotyping batch. We also included binary (yes/no) variables for maternal smoking and alcohol consumption at each timepoint. The model included a genetic relationship matrix to model cryptic relatedness and population structure. 8](#_Toc156296570)

[Supplementary Figure 3. Effect estimates and 95% confidence intervals (CI) from the linear mixed model observational analyses assessing the relationship between both maternal and paternal coffee consumption exposure (cups/day) and offspring neurodevelopmental difficulties (ND) outcomes (rank-based inverse normal transformed), while adjusting for maternal coffee consumption before pregnancy. The linear mixed models adjusted for maternal coffee consumption before pregnancy, as well as offspring birth year, maternal age at birth, paternal age at birth, maternal smoking before pregnancy, at week 15, and week 30, maternal alcohol before pregnancy, at week 15, week 24, and week 30, maternal and paternal income, maternal and paternal education, and offspring genotyping batch. We also included binary (yes/no) variables for maternal smoking and alcohol consumption at each timepoint. The model also included a genetic relationship matrix to model cryptic relatedness and population structure. 10](#_Toc156296571)

[Supplementary Materials 4. Power Calculations 13](#_Toc156296572)

[Supplementary Figure 4. Power calculations for the two sample Mendelian randomisation analyses. The solid red line represents 80% power, whereas the dashed and dotted grey lines represent different proportions of variance explained for the asssociation between the PGS and the exposure variable (R^2^(XZ)). Dashed line R^2^(xz) = 1.94%, wheras the dotted line R^2^(xz) = 0.32%. In both scenarios, other variables were kept constant (N = 46,245; alpha = 0.05; var(x) = 1.69; var(y) = 1). 13](#_Toc156296573)

[Supplementary Figure 5. Power calculations for individual level PGS analyses in mother-child duos (N = 46,245; alpha = 0.05). The solid red line represents 80% power. 14](#_Toc156296574)

[Supplementary Materials 5. Traditional observational analyses that adjust for additional potential confounders 15](#_Toc156296575)

[Supplementary Figure 6. Effect estimates and 95% confidence intervals (CI) from the traditional observational analyses assessing the relationship between both maternal and paternal coffee consumption exposure (cups/day) and offspring neurodevelopmental difficulties (ND) outcomes (rank-based inverse normal transformed) using linear regression. The models adjusted for offspring birth year, maternal age at birth, paternal age at birth, maternal smoking before pregnancy, at week 15, and week 30, maternal alcohol before pregnancy, at week 15, week 24, and week 30, maternal and paternal income, and both maternal and paternal education. We also included binary (yes/no) variables for maternal smoking and alcohol consumption at each timepoint 15](#_Toc156296576)

[Supplementary Materials 6. Traditional observational analyses that adjust for maternal coffee consumption before pregnancy 17](#_Toc156296577)

[Supplementary Figure 7. Effect estimates and 95% confidence intervals (CI) from the traditional observational analyses assessing the relationship between both maternal and paternal coffee consumption exposure (cups/day) and offspring neurodevelopmental difficulties (ND) outcomes (rank-based inverse normal transformed), while adjusting for maternal coffee consumption before pregnancy. Analyses were conducted using linear regression. The models adjusted for maternal coffee consumption before pregnancy, as well as offspring birth year, maternal age at birth, paternal age at birth, maternal smoking before pregnancy, at week 15, and week 30, maternal alcohol before pregnancy, at week 15, week 24, and week 30, maternal and paternal income, and both maternal and paternal education. We also included binary (yes/no) variables for maternal smoking and alcohol consumption at each timepoint 17](#_Toc156296578)

[Supplementary Materials 7. Collider Bias in Gene by Environment Mendelian Randomisation Analyses 19](#_Toc156296579)

[Supplementary Figure 8. Diagram illustrating a potential case of collider bias in a gene x environment Mendelian randomisation (GxE MR) analysis. Collider bias may be introduced into a GxE MR study when both A) an additional confounder (E) is related to both coffee consumption (ever/never) and the offspring outcome; and B) the SNP used to proxy the exposure (cups/day) are also related to consumption (ever/never). When conditioning on the collider (i.e. consumption) in the GxE MR framework, a pathway may open up between the SNP and the outcome through the confounder E, manifesting as a false positive association between the exposure and outcome 19](#_Toc156296580)

[Supplementary References 20](#_Toc156296581)

#

# Supplementary Materials 1. Descriptions of offspring neurodevelopmental difficulties

***Social Communication Questionnaire*** (SCQ; Rutter et al., 2003): The SCQ is a parental-report questionnaire designed to identify signs of autism, which was completed by MoBa mothers when the child was 3 and 8 years of age. At both ages, the SCQ consists of the same 40 items (yes/no response format with some items reverse-coded) relating to social and communication impairments (SCI) and restricted and repetitive behaviours (RRB). Higher scores are indicative of atypical behaviour (i.e., scored as 1 for atypical behavior and 0 for absence of atypical behavior/typical behavior.

***Child Behaviour Checklist*** (CBCL; Achenbach, 1992): The CBCL was developed to identify problem behaviour in children. The Diagnostic and Statistical Manual of Mental Disorders (DSM) oriented subscale for ADHD behaviors was used at age 18 months, 3 years and 5 years, including 4, 6 and 6 items, respectively. The items were rated by the mothers on a 3-point scale (0 = not true; 1 = somewhat or sometimes true; 2 = very true or often true), where higher scores indicate more ADHD-associated behaviors.

***Rating Scale for Disruptive Behaviour Disorders*** (RS-DBD ;Silva et al., 2005): The RS-DBD is based upon DSM-IV items and consists of 34 items relating to Oppositional Defiant Disorder, Conduct Disorder and ADHD. The 18 items related to ADHD were further divided into the subscales of inattention (9 items) and hyperactivity (9 items). Mothers evaluated their child’s behaviour at age 8 years on a 4-point scale (0 = never or seldom; 1 = sometimes; 2 = often; 3 = very often), where high scores reflect more inattentive/hyperactive behaviours.

***Conners Parent Rating Scale-Revised (Short Form)*** (CPRS-R (S); Conners et al., 1998): The CPRS-R (S) is a questionnaire used to obtain parental reports of childhood behaviour problems. The version administered in the MoBa questionnaire at child age 5 years consists of 12 items relating to inattention, hyperactivity and impulsivity. Responses were scored on a 4-point Likert scale (0 = not true/never/seldom; 1 = somewhat true/sometimes; 2 = quite often; 3 = very often), where increased scores represent increased behavioural problems.

***Ages and Stages Questionnaire*** (ASQ; Richter & Janson, 2007; Squires et al., 1999): The ASQ is part of an age-specific series of questionnaires for child development, with subscales relating to communication, gross motor, fine motor, problem solving and personal-social. The ASQ was included in the 18 months, 3 years and 5 years MoBa Questionnaires and parents answer ‘yes’, ‘sometimes’ or ‘not yet’.

***The Children’s Communication Checklist-2 Short Scale*** (CCC-S; Bishop, 2003; Norbury et al., 2004): The CCC-S is a 13-item short scale of the CCC-2 that can identify children with potential speech, language and communication needs. Parents answer ‘never or rarely’, ‘sometimes’, ‘often’ or ‘very often’.

***Child Development Inventory*** (CDI; Chaffee et al., 1990; Ireton, 1992): The CDI was designed to assess childhood development from ages 15 months to 6 years, and was included in the 5-year MoBa Questionnaire. This gross- and fine motor skill subscale consists of 12 yes/no questions, 10 of which are derived from the 1992 iteration of the CDI, while the other two items were designed specifically for MoBa. The CDI was reverse coded so that increased scores reflect greater motor difficulties.

# Supplementary Materials 2. Observational analyses using genetic linear mixed models.

An additional genetic linear mixed model approach, which is more robust to the effects of cryptic relatedness and population structure, was used to explore the relationship between maternal coffee consumption and offspring NDs in the genotyped MoBa sample. To our awareness, this approach has not been employed in previous observational studies of maternal caffeine intake and offspring neurodevelopmental difficulties, including the past study involving MoBa^11^.

Similar models to those in our traditional epidemiological analyses were used, except now the genetic linear mixed model included GRMs (generated from all genotyped and imputed autosomal loci) in the random effects part of the model. The first model investigated the relationship between the exposure, maternal or paternal coffee consumption, and offspring NDs while adjusting for offspring birth year, parental ages at birth, and offspring genotyping batch as fixed effects. The second model also included these fixed effects as covariates (offspring birth year, parental ages at birth, offspring genotyping batch) but also modelled additional potential confounders as covariates (parental education and income, continuous measures of maternal smoking and alcohol consumption, and binary variables for maternal smoking and alcohol consumption). The last model attempted to isolate the intrauterine effect of maternal coffee consumption, by including maternal coffee consumption before pregnancy as a covariate alongside the aforementioned covariates. The genetic linear mixed model analyses were conducted using the GCTA software tool v 1.93.2 beta^12,13^.

**Supplementary Figure 1.** Effect estimates and 95% confidence intervals (CI) from the observational analyses assessing the relationship between both maternal and paternal coffee consumption exposure (cups/day) and offspring neurodevelopmental difficulties (ND) outcomes (rank-based inverse normal transformed) using the linear mixed model approach. The linear mixed model was used to assess the relationship between maternal or paternal coffee consumption at each timepoint and offspring NDs, while adjusting for offspring birth year, maternal age at birth, paternal age at birth, and offspring genotyping batch. The model also included a genetic relationship matrix to model cryptic relatedness and population structure. SCQ-full: Social Communication Questionnaire; SCQ-RRB: Social Communication Questionnaire restricted and repetitive behaviour subscale; SCQ-SCI: Social Communication Questionnaire social communication impairment subscale; CBCL-ADHD: Child Behaviour Checklist ADHD subscale; RS-DBD-ADHD: Rating Scale for Disruptive Behaviour Disorders ADHD subscale; RS-DBD-INA: Rating Scale for Disruptive Behaviour Disorders inattention subscale; RS-DBD-HYP: Rating Scale for Disruptive Behaviour Disorders hyperactivity subscale; CPRS: Conners Parent Rating Scale-Revised short form; ASQ-LANG: Ages and Stages Questionnaire language subscale; CCC-S: The Children’s Communication Checklist-2 Short Scale; ASQ-MOTOR: Ages and Stages Questionnaire motor items; CDI-MOTOR: Child Development Inventory motor subscale.

**
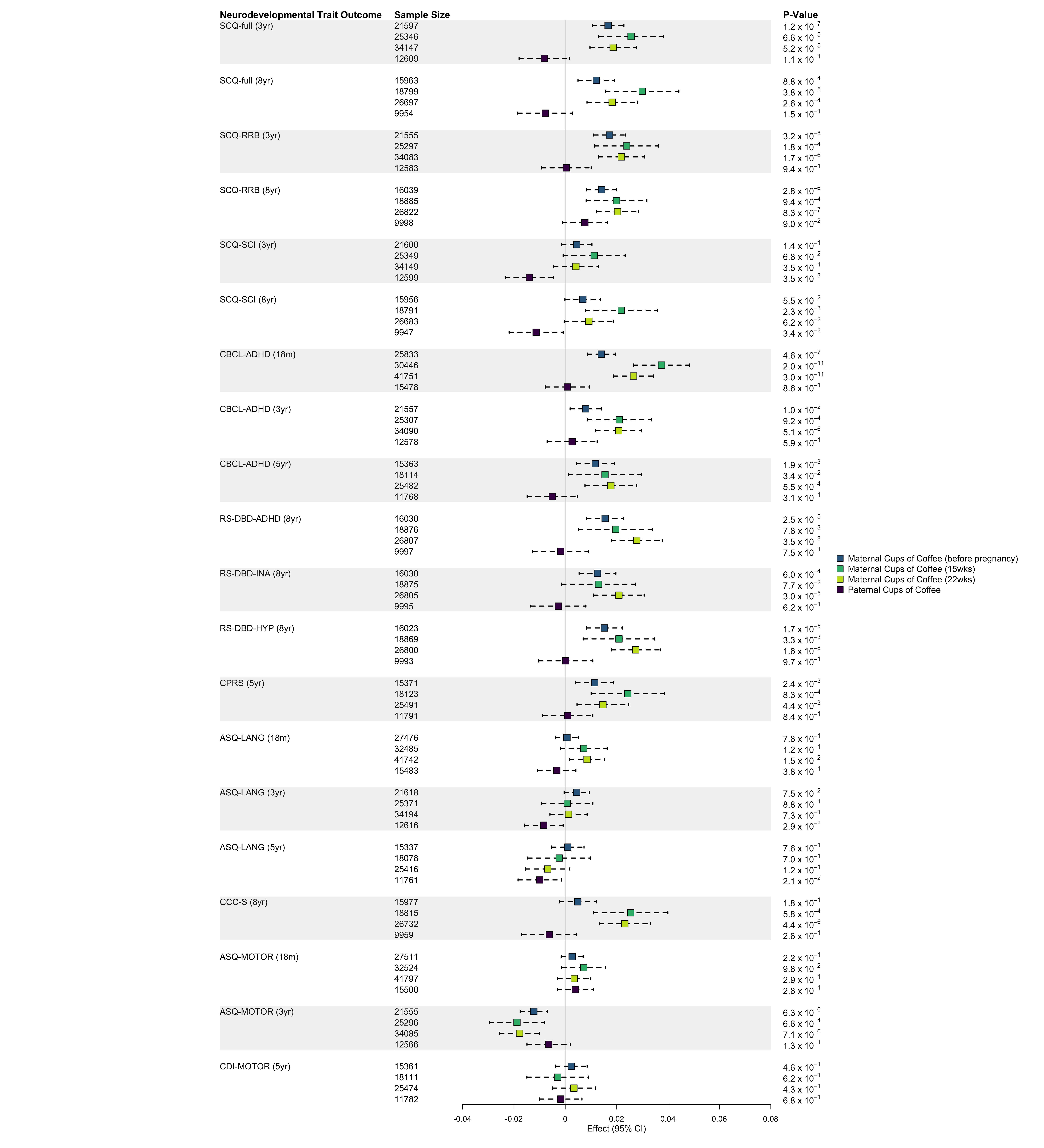
**

## **Supplementary Figure 2.** Effect estimates and 95% confidence intervals (CI) from the traditional observational analyses that adjusted for additional confounders assessing the relationship between both maternal and paternal coffee consumption exposure (cups/day) and offspring neurodevelopmental difficulties (ND) outcomes (rank-based inverse normal transformed). The linear mixed models adjusted for offspring birth year, maternal age at birth, paternal age at birth, maternal smoking before pregnancy, at week 15, and week 30, maternal alcohol before pregnancy, at week 15, week 24, and week 30, maternal and paternal income, maternal and paternal education, and offspring genotyping batch. We also included binary (yes/no) variables for maternal smoking and alcohol consumption at each timepoint. The model included a genetic relationship matrix to model cryptic relatedness and population structure. SCQ-full: Social Communication Questionnaire; SCQ-RRB: Social Communication Questionnaire restricted and repetitive behaviour subscale; SCQ-SCI: Social Communication Questionnaire social communication impairment subscale; CBCL-ADHD: Child Behaviour Checklist ADHD subscale; RS-DBD-ADHD: Rating Scale for Disruptive Behaviour Disorders ADHD subscale; RS-DBD-INA: Rating Scale for Disruptive Behaviour Disorders inattention subscale; RS-DBD-HYP: Rating Scale for Disruptive Behaviour Disorders hyperactivity subscale; CPRS: Conners Parent Rating Scale-Revised short form; ASQ-LANG: Ages and Stages Questionnaire language subscale; CCC-S: The Children’s Communication Checklist-2 Short Scale; ASQ-MOTOR: Ages and Stages Questionnaire motor items; CDI-MOTOR: Child Development Inventory motor subscale.


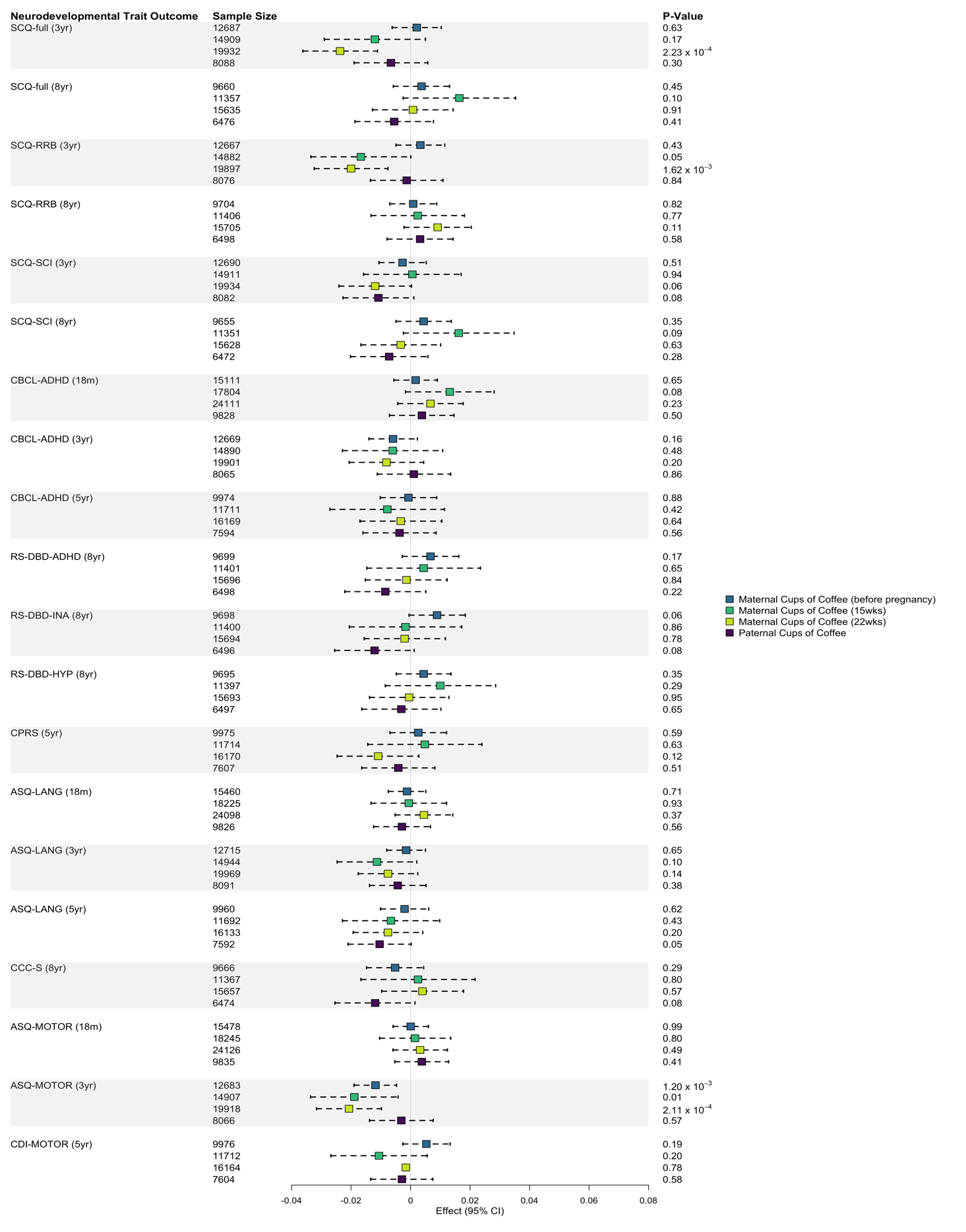

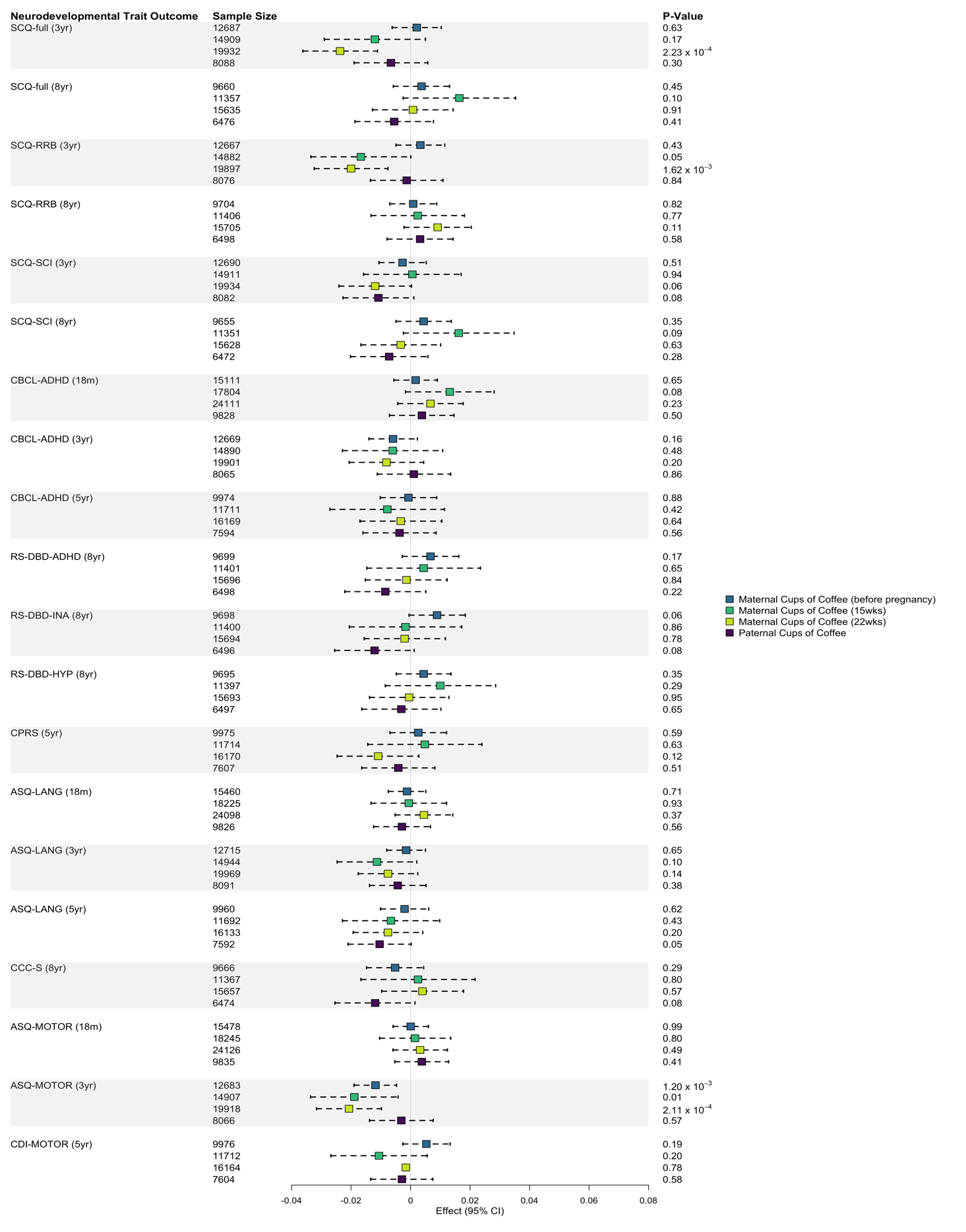


## **Supplementary Figure 3.** Effect estimates and 95% confidence intervals (CI) from the linear mixed model observational analyses assessing the relationship between both maternal and paternal coffee consumption exposure (cups/day) and offspring neurodevelopmental difficulties (ND) outcomes (rank-based inverse normal transformed), while adjusting for maternal coffee consumption before pregnancy. The linear mixed models adjusted for maternal coffee consumption before pregnancy, as well as offspring birth year, maternal age at birth, paternal age at birth, maternal smoking before pregnancy, at week 15, and week 30, maternal alcohol before pregnancy, at week 15, week 24, and week 30, maternal and paternal income, maternal and paternal education, and offspring genotyping batch. We also included binary (yes/no) variables for maternal smoking and alcohol consumption at each timepoint. The model also included a genetic relationship matrix to model cryptic relatedness and population structure. SCQ-full: Social Communication Questionnaire; SCQ-RRB: Social Communication Questionnaire restricted and repetitive behaviour subscale; SCQ-SCI: Social Communication Questionnaire social communication impairment subscale; CBCL-ADHD: Child Behaviour Checklist ADHD subscale; RS-DBD-ADHD: Rating Scale for Disruptive Behaviour Disorders ADHD subscale; RS-DBD-INA: Rating Scale for Disruptive Behaviour Disorders inattention subscale; RS-DBD-HYP: Rating Scale for Disruptive Behaviour Disorders hyperactivity subscale; CPRS: Conners Parent Rating Scale-Revised short form; ASQ-LANG: Ages and Stages Questionnaire language subscale; CCC-S: The Children’s Communication Checklist-2 Short Scale; ASQ-MOTOR: Ages and Stages Questionnaire motor items; CDI-MOTOR: Child Development Inventory motor subscale.


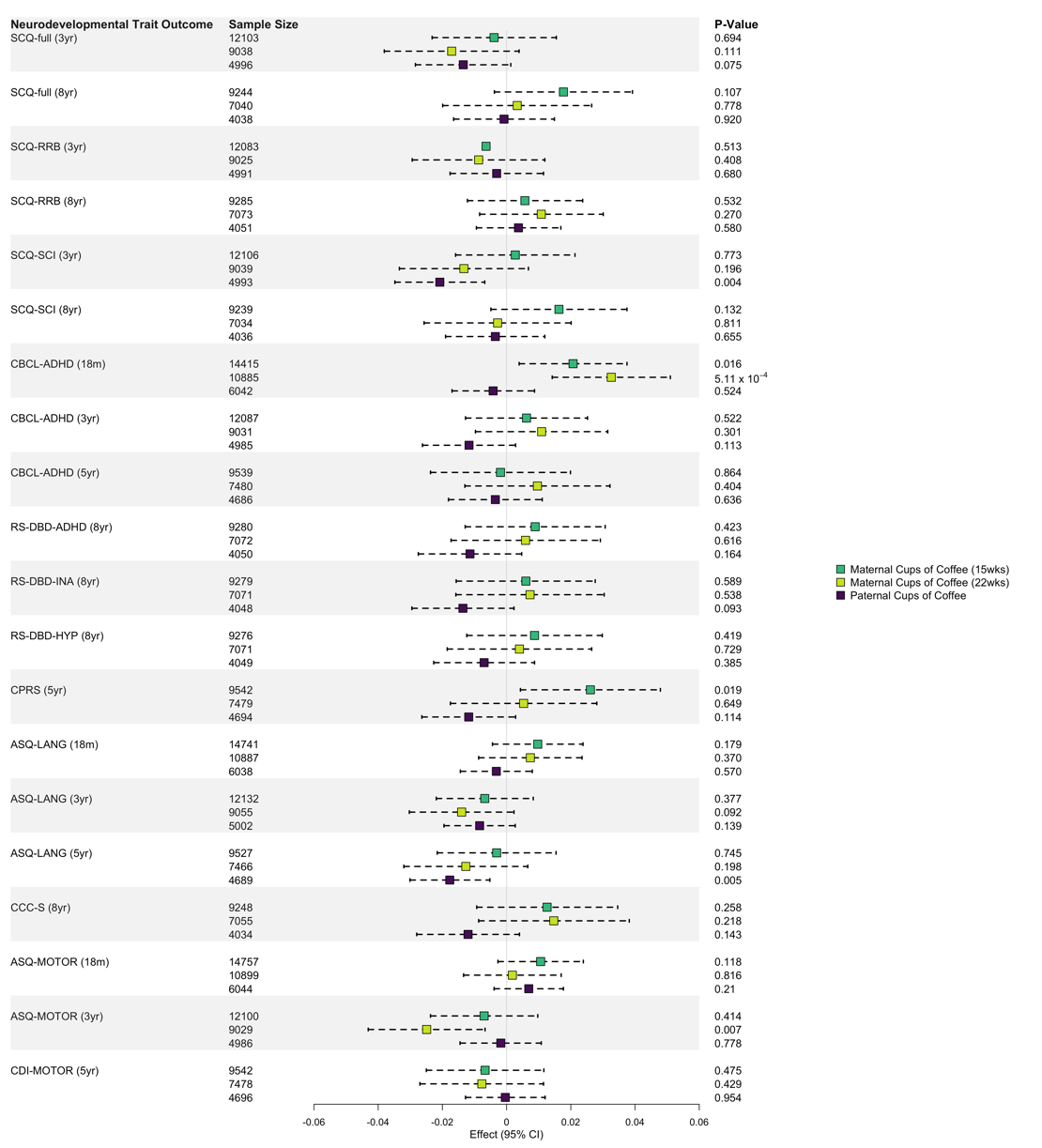

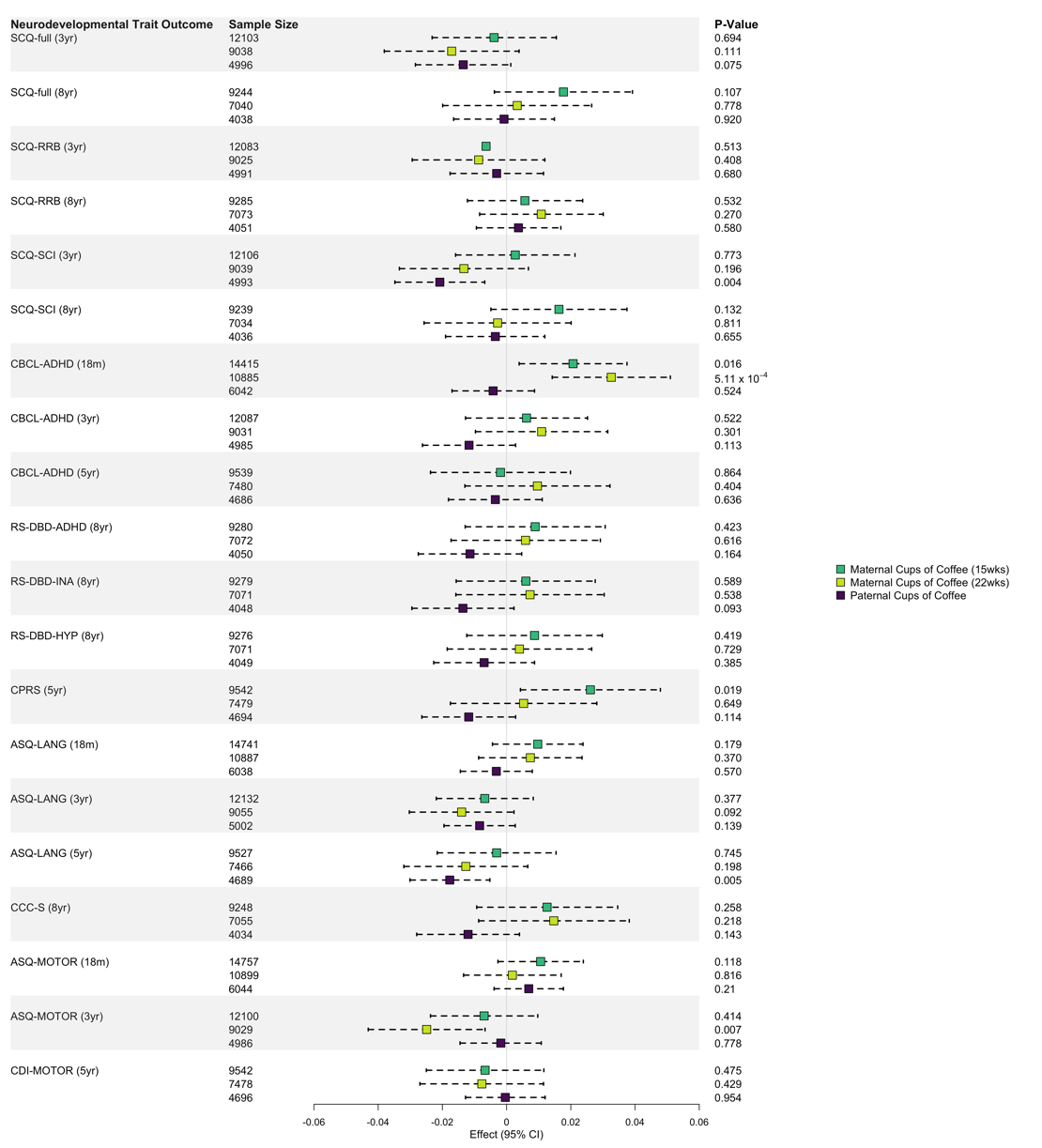


The unadjusted genetic linear mixed model analyses (i.e. not adjusting for smoking/alcohol/education) also found strong evidence for positive associations between maternal coffee consumption and many offspring difficulties with social communication and behavioural flexibility, and attention and hyperactive-impulsive behaviour trait scores (Supplementary Figure 1; p < 0.005). There was less evidence for association with language difficulties (Supplementary Figure 1; p < 0.005; only CCC-S-8yr at week 15 and 22). Negative correlations were observed between coffee consumption and ASQ-MOTOR-3yr across all time points, however no effect was observed on ASQ-MOTOR-18m and CDI-MOTOR-5yr. The negative control analyses found little evidence that paternal coffee consumption was associated with the offspring NDs, except for SCQ-SCI-3yr (p < 0.005; negative effect).

When adjusting for education, income, smoking and alcohol related variables, there was less evidence for an effect of maternal coffee consumption, apart from a few negative associations (Supplementary Figure 2; p < 0.005; SCQ-full-3yr at week 22, ASQ-MOTOR-3yr before pregnancy and at week 22).

When adjusting for maternal coffee consumption before pregnancy, in most cases, the confidence intervals are overlapping with zero (Supplementary Figure 3). The only significant (p < 0.005) results were a positive association between maternal coffee consumption at week 22 and CBCL-ADHD-18m, and a negative association between paternal coffee consumption and SCQ-SCI-3yr.

Only minor differences were observed between the results from the genetic linear mixed model approach conducted in the MoBa genotyped sample and the traditional observational analyses conducted in the full sample. While this approach is more robust to the effects of cryptic relatedness and population structure due to the incorporation of a GRM, notably, the genotyped sample is more selected than the full MoBa cohort.

# Supplementary Materials 3. Power Calculations

We used the MR power calculator (<https://shiny.cnsgenomics.com/mRnd/>)^14^ to approximate the size of the causal effect that we had 80% statistical power to detect in our Two Sample MR analyses. We calculated power twice, the first time we set the proportion of variance explained for the association between the weighted PGS and the exposure variable to 1.94% (according to the effect estimates reported in the exposure GWAS), and a second time we used internal estimates (ST5) of the proportion of variance explained in the exposure by the weighted PGS (0.32%) from linear regression analyses in R. Although the MR power calculator is designed for one-sample MR analyses, it has been used to provide an approximation of power in the present Two-Sample MR study. We also used the Maternal and Offspring Genetic Effects Power Calculator (<https://evansgroup.di.uq.edu.au/MGPC/>) to estimate what maternal genetic effect we had 80% power to detect in the mother-child duo PGS analyses^15^. For all power calculations, we assumed a (two sided) type 1 error rate of α = 0.05.

We had 80% power to detect an approximate causal effect size of 0.072 and 0.177 (i.e. change in our rank-based inverse normal transformed NDs per cup of coffee per day; Supplementary Figure 4) when the proportion of variance explained in the exposure by the PGS is 1.94% and 0.32%, respectively (α = 0.05). Our maternal genetic effect power calculations found that we had 80% power to detect a maternal genetic effect that explained 0.023% of the variance in the outcome (α = 0.05; Supplementary Figure 5).

## **Supplementary Figure 4.** Power calculations for the two sample Mendelian randomisation analyses. The solid red line represents 80% power, whereas the dashed and dotted grey lines represent different proportions of variance explained for the asssociation between the PGS and the exposure variable (R^2^(XZ)). Dashed line R^2^(xz) = 1.94%, wheras the dotted line R^2^(xz) = 0.32%. In both scenarios, other variables were kept constant (N = 46,245; alpha = 0.05; var(x) = 1.69; var(y) = 1).

## **Supplementary Figure 5.** Power calculations for individual level PGS analyses in mother-child duos (N = 46,245; alpha = 0.05). The solid red line represents 80% power.

# Supplementary Materials 4. Traditional observational analyses that adjust for additional potential confounders

**Supplementary Figure 6.** Effect estimates and 95% confidence intervals (CI) from the traditional observational analyses assessing the relationship between both maternal and paternal coffee consumption exposure (cups/day) and offspring neurodevelopmental difficulties (ND) outcomes (rank-based inverse normal transformed) using linear regression. The models adjusted for offspring birth year, maternal age at birth, paternal age at birth, maternal smoking before pregnancy, at week 15, and week 30, maternal alcohol before pregnancy, at week 15, week 24, and week 30, maternal and paternal income, and both maternal and paternal education. We also included binary (yes/no) variables for maternal smoking and alcohol consumption at each timepoint. SCQ-full: Social Communication Questionnaire; SCQ-RRB: Social Communication Questionnaire restricted and repetitive behaviour subscale; SCQ-SCI: Social Communication Questionnaire social communication impairment subscale; CBCL-ADHD: Child Behaviour Checklist ADHD subscale; RS-DBD-ADHD: Rating Scale for Disruptive Behaviour Disorders ADHD subscale; RS-DBD-INA: Rating Scale for Disruptive Behaviour Disorders inattention subscale; RS-DBD-HYP: Rating Scale for Disruptive Behaviour Disorders hyperactivity subscale; CPRS: Conners Parent Rating Scale-Revised short form; ASQ-LANG: Ages and Stages Questionnaire language subscale; CCC-S: The Children’s Communication Checklist-2 Short Scale; ASQ-MOTOR: Ages and Stages Questionnaire motor items; CDI-MOTOR: Child Development Inventory motor subscale.


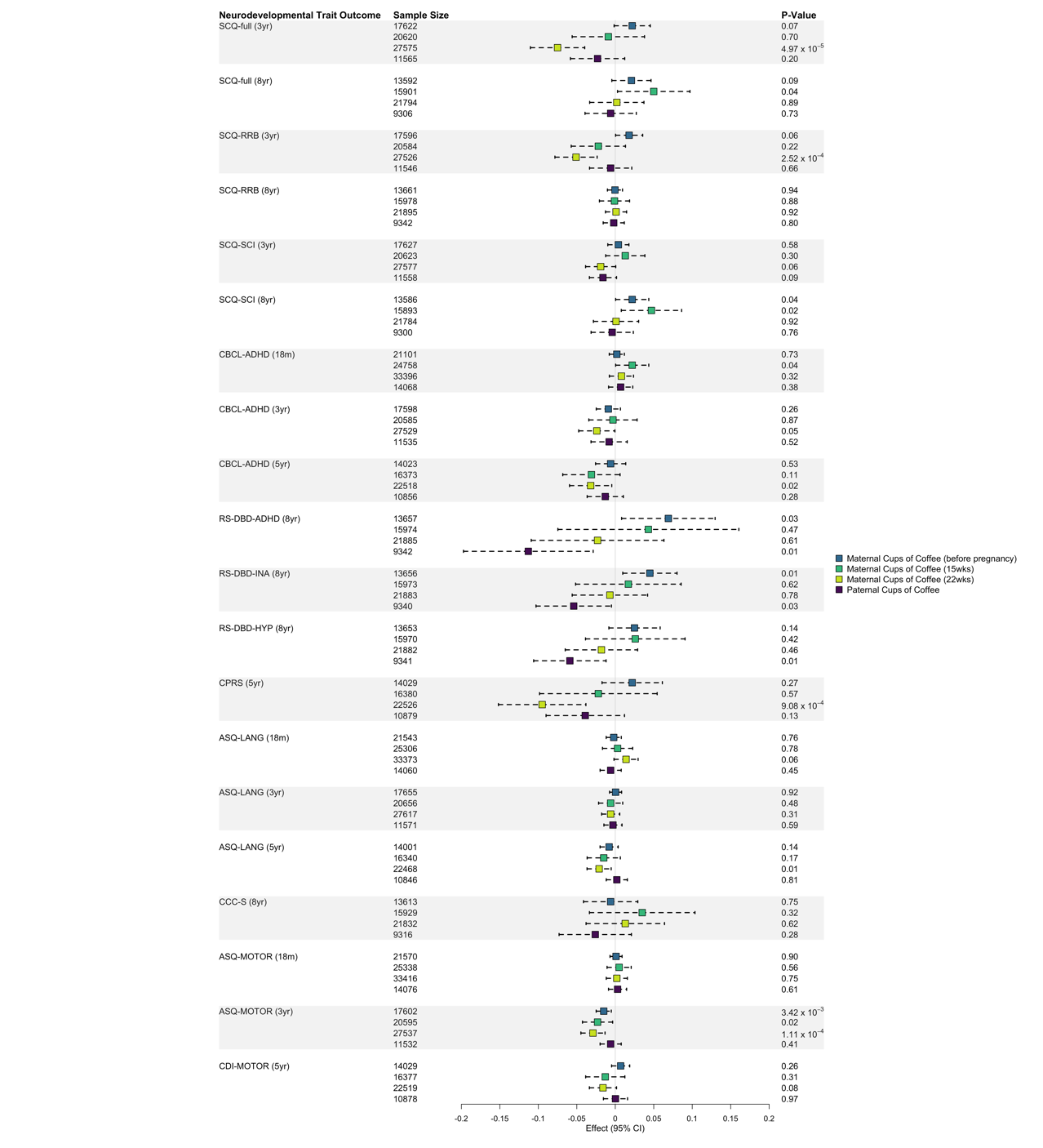


# Supplementary Materials 5. Traditional observational analyses that adjust for maternal coffee consumption before pregnancy

**Supplementary Figure 7.** Effect estimates and 95% confidence intervals (CI) from the traditional observational analyses assessing the relationship between both maternal and paternal coffee consumption exposure (cups/day) and offspring neurodevelopmental difficulties (ND) outcomes (rank-based inverse normal transformed), while adjusting for maternal coffee consumption before pregnancy. Analyses were conducted using linear regression. The models adjusted for maternal coffee consumption before pregnancy, as well as offspring birth year, maternal age at birth, paternal age at birth, maternal smoking before pregnancy, at week 15, and week 30, maternal alcohol before pregnancy, at week 15, week 24, and week 30, maternal and paternal income, and both maternal and paternal education. We also included binary (yes/no) variables for maternal smoking and alcohol consumption at each timepoint. SCQ-full: Social Communication Questionnaire; SCQ-RRB: Social Communication Questionnaire restricted and repetitive behaviour subscale; SCQ-SCI: Social Communication Questionnaire social communication impairment subscale; CBCL-ADHD: Child Behaviour Checklist ADHD subscale; RS-DBD-ADHD: Rating Scale for Disruptive Behaviour Disorders ADHD subscale; RS-DBD-INA: Rating Scale for Disruptive Behaviour Disorders inattention subscale; RS-DBD-HYP: Rating Scale for Disruptive Behaviour Disorders hyperactivity subscale; CPRS: Conners Parent Rating Scale-Revised short form; ASQ-LANG: Ages and Stages Questionnaire language subscale; CCC-S: The Children’s Communication Checklist-2 Short Scale; ASQ-MOTOR: Ages and Stages Questionnaire motor items; CDI-MOTOR: Child Development Inventory motor subscale.


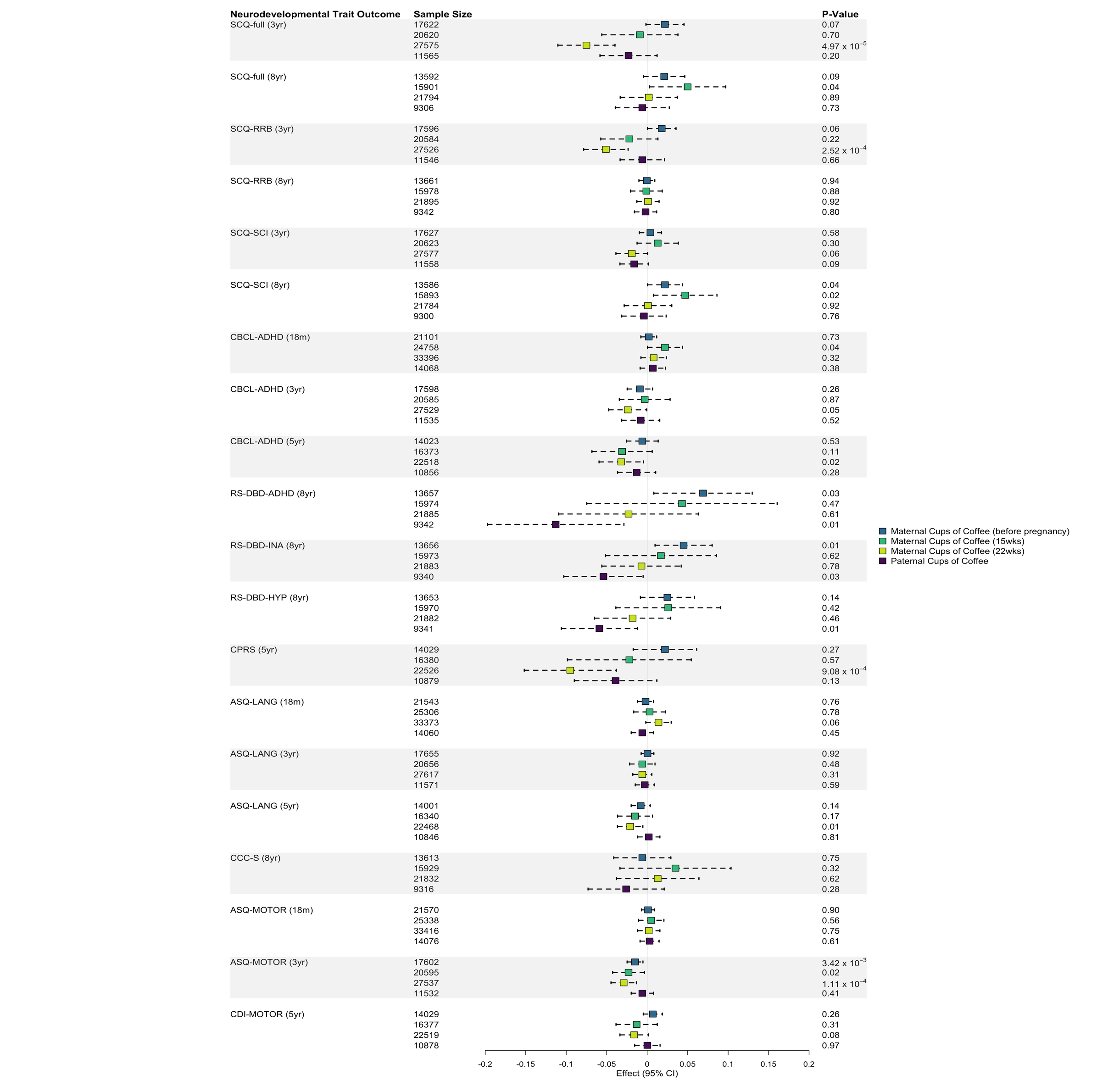

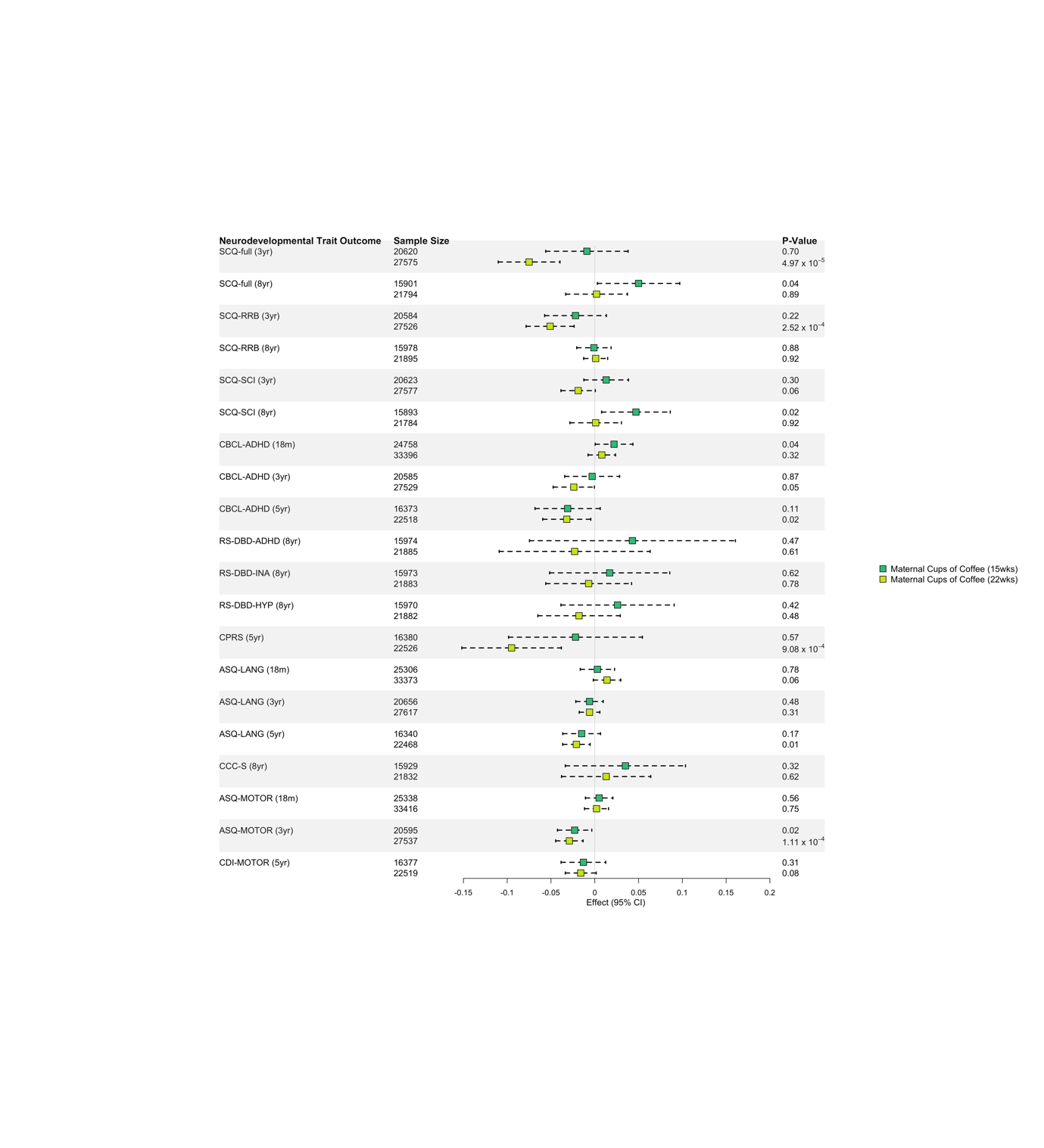


# Supplementary Materials 6. Collider Bias in Gene by Environment Mendelian Randomisation Analyses

**Supplementary Figure 8**. Diagram illustrating a potential case of collider bias in a gene x environment Mendelian randomisation (GxE MR) analysis. Collider bias may be introduced into a GxE MR study when both A) an additional confounder (E) is related to both coffee consumption (ever/never) and the offspring outcome; and B) the SNP used to proxy the exposure (cups/day) are also related to consumption (ever/never). When conditioning on the collider (i.e. consumption) in the GxE MR framework, a pathway may open up between the SNP and the outcome through the confounder E, manifesting as a false positive association between the exposure and outcome.


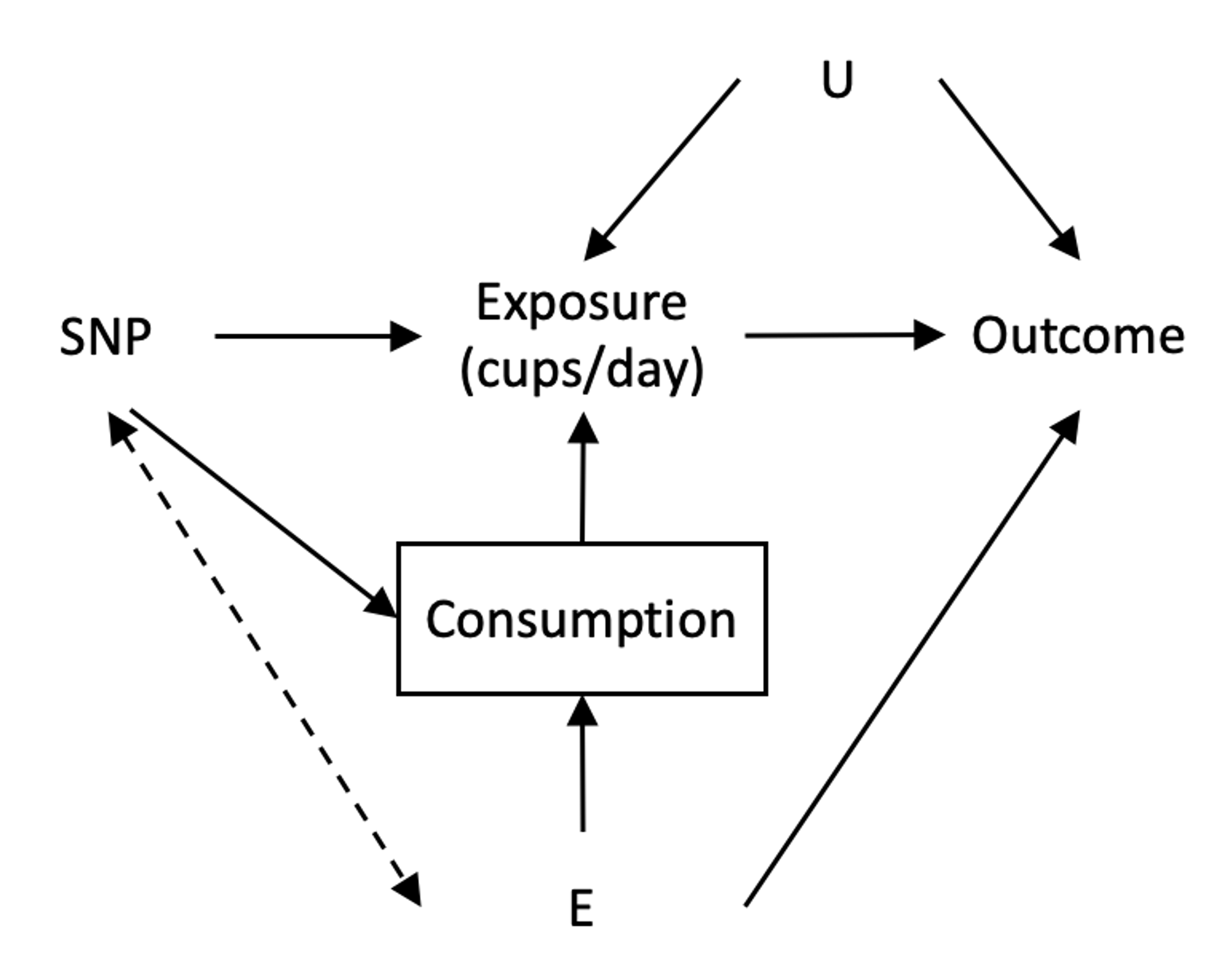


# Supplementary References

1. Rutter M, Bailey A, Lord C. The social communication questionnaire: manual. Torrance, CA: WPS. 2003;

2. Achenbach TM. Manual for the child behavior checklist/2-3 and 1992 profile. Burlington, VT: Dept. of Psychiatry, University of Vermont; 1992.

3. Silva RR, Alpert M, Pouget E, et al. A Rating Scale for Disruptive Behavior Disorders, Based on the DSM-IV Item Pool. Psychiatric Quarterly [Internet]. 2005;**76**(4):327–339. Available from: https://doi.org/10.1007/s11126-005-4966-x

4. Conners CK, Sitarenios G, Parker JDA, Epstein JN. The Revised Conners’ Parent Rating Scale (CPRS-R): Factor Structure, Reliability, and Criterion Validity. J Abnorm Child Psychol [Internet]. 1998;**26**(4):257–268. Available from: https://doi.org/10.1023/A:1022602400621

5. Richter J, Janson H. A validation study of the Norwegian version of the Ages and Stages Questionnaires. Acta Paediatr [Internet]. 2007 [cited 2022 Dec 8];**96**(5):748–752. Available from: https://www.academia.edu/25490194/A_validation_study_of_the_Norwegian_version_of_the_Ages_and_Stages_Questionnaires

6. Squires J, Bricker DD, Potter L. The ASQ user’s guide. 2nd ed. Baltimore, MD: Paul H. Brookes; 1999.

7. Norbury CF, Nash M, Baird G, Bishop DVM. Using a parental checklist to identify diagnostic groups in children with communication impairment: a validation of the Children’s Communication Checklist—2. Int J Lang Commun Disord. Taylor & Francis; 2004;**39**(3):345–364.

8. Bishop DVM. The Children’s communication checklist. Psychological Corporation London; 2003.

9. Ireton H. The Child Development Inventory Manual. Minneapolis, MN; 1992.

10. Chaffee CA, Cunningham CE, Secord-Gilbert M, Elbard H, Richards J. Screening effectiveness of the Minnesota Child Development Inventory expressive and receptive language scales: Sensitivity, specificity, and predictive value. Psychological Assessment: A Journal of Consulting and Clinical Psychology. American Psychological Association; 1990;**2**(1):80.

11. Berglundh S, Vollrath M, Brantsæter AL, et al. Maternal caffeine intake during pregnancy and child neurodevelopment up to eight years of age—Results from the Norwegian Mother, Father and Child Cohort Study. Eur J Nutr. Springer; 2021;**60**:791–805.

12. Yang J, Lee SH, Goddard ME, Visscher PM. GCTA: A Tool for Genome-wide Complex Trait Analysis. The American Journal of Human Genetics [Internet]. 2011;**88**(1):76–82. Available from: https://www.sciencedirect.com/science/article/pii/S0002929710005987

13. Yang J, Benyamin B, McEvoy BP, et al. Common SNPs explain a large proportion of the heritability for human height. Nat Genet [Internet]. Nature Publishing Group, a division of Macmillan Publishers Limited. All Rights Reserved.; 2010 Jun 20;**42**:565. Available from: https://doi.org/10.1038/ng.608

14. Brion M-JA, Shakhbazov K, Visscher PM. Calculating statistical power in Mendelian randomization studies. Int J Epidemiol [Internet]. 2013 Oct 1;**42**(5):1497–1501. Available from: https://doi.org/10.1093/ije/dyt179

15. Moen G-H, Hemani G, Warrington NM, Evans DM. Calculating Power to Detect Maternal and Offspring Genetic Effects in Genetic Association Studies. Behav Genet [Internet]. 2019;**49**(3):327–339. Available from: https://doi.org/10.1007/s10519-018-9944-9
